# Stabilization of norovirus GII.3 virus-like particles by rational disulfide engineering

**DOI:** 10.1038/s41541-025-01254-2

**Published:** 2025-08-19

**Authors:** Christopher Warren, Jennifer D. Galli, Karin Bystol, Gregory O’Donnell, Andrew R. Swartz, Emily A. Dewar, Corey May Fulton, Pamela Shen, Estibaliz Gonzalez-Fernandez, Lizzy Aurora DeWitt, Uijin Jeong, Oscar Chi-Chien Pan, Sean Miller, Arthur Fridman, Courtney David, Zhifeng Chen, Jiajie Wei

**Affiliations:** 1 Infectious Diseases and Vaccines Discovery, Merck & Co., Inc., West Point, PA USA; 2 Quantitative Biosciences, Merck & Co., Inc., West Point, PA USA; 3 Process Research & Development, Merck & Co., Inc., West Point, PA USA; 4 Analytical Research and Development, Merck & Co., Inc, West Point, PA USA; 5 Data Science and Scientific Informatics, RaDS-IT, Merck & Co., Inc., Rahway, NJ USA; 6 Discovery Pharmaceutical Sciences, Merck & Co., Inc., West Point, PA USA

**Keywords:** Infectious diseases, Vaccines

## Abstract

Noroviruses are non-enveloped, single-stranded positive-sense RNA viruses and the leading cause of gastroenteritis worldwide. The major capsid protein, VP1, can self-assemble into non-infectious virus-like particles (VLPs), representing an attractive vaccine platform. It was demonstrated that engineered disulfide bonds within VP1 could significantly stabilize VLPs of the archetypal GI.1 strain. Here, we apply a similar strategy to VLPs of multiple circulating GII genotypes. We find that engineered disulfide mutations can significantly stabilize VLPs of the GII.3 strain, but not the closely related GII.6 strain. Disulfide-stabilized GII.3 VLPs (GII.3-DS1) exhibit increased yields, greater homogeneity, and higher thermal stability compared to wild-type GII.3 VLPs. GII.3-DS1 VLPs are a superior reagent in immunological assays compared to the wild-type counterpart. Importantly, mRNA encoding GII.3-DS1 elicits superior humoral immune responses compared to wild-type GII.3 mRNA in mice. These results demonstrate the utility of rational VLP stabilization for advancing vaccine development efforts.

## Introduction

Noroviruses are a leading cause of acute gastroenteritis (AGE) across all age groups and are responsible for 18% of diarrheal disease globally^1^. Norovirus is highly contagious and transmitted by the fecal-oral route^2–4^. Infections have a broad clinical spectrum that ranges from asymptomatic or self-limiting illness to severe or fatal disease. The public health impact generated by noroviruses is significant, with more than 20 million cases in the United States, 680 million cases worldwide and 219 thousand deaths annually. The disease burden is especially significant among children, with more than 200 million infections reported in children under 5 years of age and 50,000 cases resulting in death every year^5–7^. The total economic burden of noroviruses is estimated to be $64.5 billion annually^5^. Despite the prevalence of norovirus infection and associated disease burden, there are no approved prophylactics or targeted therapies. Treatments remain only palliative, resulting in a significant unmet medical need.

Noroviruses are non-enveloped, single-stranded RNA viruses belonging to the *Caliciviridae* family. Noroviruses are classified into 10 genogroups (GI-GX) and 48 genotypes^8^. The GII genogroup is responsible for 90% of global outbreaks, with GII.4 at greater than 50% in all age groups since the mid-1990s^9^. While GII.4 is most common among all age groups, GII.3 and GII.6 are one of the most common genotypes infecting children and infants^10–13^. Based on data from NoroSurv, a global network for norovirus strain surveillance among children, from January 2015 to March 2025, 15% and 6% of submitted sequences were typed as GII.3 and GII.6, respectively^13,14^.

Human noroviruses contain three overlapping open reading frames (ORF) encoding 6 non-structural proteins and 2 structural proteins, including major capsid protein VP1 and minor capsid protein VP2. VP1 is a 60 kDa protein that can assemble into virus-like particles (VLPs), which morphologically and antigenically mimic native virions^15–17^. VP2 is not required for virion or VLP formation but is thought to play a role in viral genome encapsidation, homogeneity and infectivity^18–20^. The norovirus capsid is primarily a *T* = 3 icosahedral shell approximately 35 to 50 nm in diameter and composed of 180 copies of VP1, organized into 90 dimers^21^. Structural studies have defined two major domains in VP1, the shell (S) and the protruding (P) domains, linked by a flexible hinge region. The P-domain can be further divided into P1 and P2 subdomains, the latter being critical for norovirus infection by interacting with carbohydrates present on various histoblood group antigens (HBGAs) in the host^15,22^.

Norovirus vaccine development has been challenging, partially due to the lack of an in vitro culture system for human norovirus. VLPs formed by VP1 mimic native virions morphologically and immunologically, representing a potential vaccine platform. VP1-based vaccine candidates, including VLPs, P particles, and recombinant adenoviruses, elicit HBGA blocking antibodies, a potential correlate of protection for norovirus infection and disease identified by human challenge studies^23–25^. In addition, clinical trial data demonstrate that VP1-based vaccines can stimulate other immunologic markers potentially associated with the protection of norovirus disease and infection, such as serum immunoglobulin G (IgG), serum immunoglobulin A (IgA), fecal IgA, salivary IgA, and norovirus-specific memory IgG B-cells^23,25,26^. Regardless of the vaccine modality, VLPs are a required reagent to support vaccine characterization and immunological assays.

Structure-guided protein design has been particularly impactful in the field of vaccines, as many viral antigens are unstable, metastable, or sample multiple distinct conformations requiring rational mutagenesis for suitable vaccine development. Structure-guided stabilization approaches have been successfully applied to multiple clinically approved vaccines, including for SARS-CoV2 and RSV. The SARS-CoV2 Spike (S) protein was stabilized in the prefusion conformation by introducing a double proline (2 P) mutation within the S2 domain^27^. These mutations were based on extensive research on the structure of the S protein of MERS^28^, allowing for the rapid development of vaccines against SARS-CoV2. Second-generation constructs containing additional proline mutations and disulfide bonds have further increased stability^29,30^. In the case of RSV, structure-guided stabilization of the prefusion conformation of the fusion (F) glycoprotein has paved the way for the recent development of multiple clinically approved RSV vaccines^31^. The basis of these RSV vaccines is the DS-Cav1 construct, which stabilized the prefusion conformation by a combination of disulfide and cavity filling mutations^32^. These foundational examples represent a new paradigm of rational stabilization of viral antigens for successful vaccine development.

Recently, it was demonstrated that a single engineered disulfide bond increased the stability of norovirus VLPs of the archetypical GI.1 strain^33^. Here, we apply a similar protein engineering strategy to VLPs of multiple currently circulating GII genotypes. We show that these design principles are not generalizable across multiple norovirus strains. Engineered disulfide mutations can significantly stabilize VLPs of the GII.3 strain, but not the closely related GII.6 strain. Disulfide stabilization of GII.3 (GII.3-DS1) results in increased VLP yield from mammalian cell expression, along with more compact and less polydisperse VLPs with significantly higher thermal stability compared to the wild-type counterpart. Negative-stain electron microscopy (nsEM) further confirms that engineered disulfides increase GII.3 VLP stability, resulting in more homogenous particles. We show that stabilized GII.3-DS1 VLPs can be recognized by human sera in ELISA assays and can be used in Histo-Blood Group Antigen (HBGA) assays to test functional antibodies against VP1. Finally, mice vaccinated with GII.3-DS1 mRNA show superior ELISA binding and HBGA blocking titers compared to those vaccinated with wild-type GII.3 mRNA. These results demonstrate the utility of rational VLP stabilization and pave the way for vaccine development efforts.

## Results

### Design and expression of disulfide-stabilized GII VLPs

**Fig. 1:**
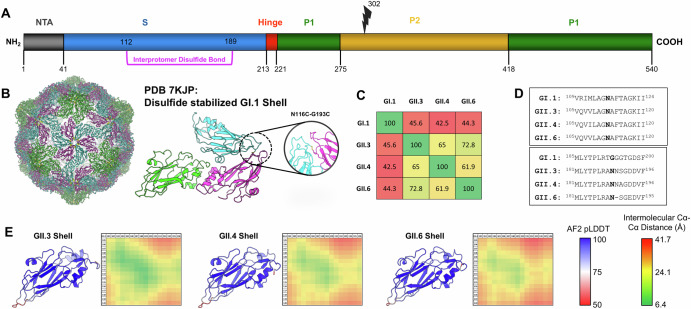
Structure-guided stabilization of norovirus VLPs. **A** Domain map of the norovirus VP1 protein showing the shell (S), hinge, and protruding (P1/P2) domains. Protease cleavage site present in GII.3 and GII.6 shown at residue 302 (lightning bolt). Engineered disulfide bond (DS1) between interprotomer residues N112 and N189 shown in purple. **B** Structure of the disulfide-stabilized GI.1 shell (PDB 7KJP) colored by chain. Left shows the intact *T* = 3 VLP. Asymmetric unit shown on the right with the interprotomer disulfide highlighted. **C** Sequence identity matrix of full-length VP1 based on the alignment of 4 norovirus genotypes. **D** Sequence alignment of VP1 from 4 norovirus genotypes in the regions of the engineered disulfide bond. Cysteine mutation positions are in bold. **E** Alphafold models of the shell domains of three GII genotypes colored by pLDDT. Monomeric models were aligned to the GI.1 *T* = 3 asymmetric unit (PDB 7KJP) and their energies were minimized. Interprotomer Ca-Ca distance matrices were calculated between loops containing residues 105–120 (*y*-axis) and 181–196 (*x*-axis) to predict proximity of residue pairs within the *T* = 3 VLP structure.

We sought to engineer a disulfide bond that could stabilize the predominant structure, *T* = 3 icosahedron, of VLPs from GII genotypes. Previous structural studies indicate that VLPs from various GII genotypes can assemble into *T* = 1, *T* = 3, and *T* = 4 icosahedrons^21^. An earlier study showed that engineering a single disulfide bond between residues 116 and 193 at the 3-fold and 5-fold symmetry axes could significantly stabilize *T* = 3 VLPs derived from the GI.1 genotype (Fig. 1B)^33^. We sought to identify analogous positions in circulating norovirus strains from the GII genotypes. Despite significant sequence variability in these regions, we identified residues 112 and 189 in genotypes GII.3, GII.4 and GII.6 as candidate sites for analogous stabilizing mutations (Fig. 1A, C, D). We next modeled the structures of the *T* = 3 shells from these genotypes using a combination of AlphaFold2 and PyRosetta, and subsequently measured intermolecular distances between 2 loops containing residues 105–120 and 181–196 (Fig. 1E)^34,35^. This structural modeling indicated that residues 112 and 189 are likely in close proximity at the 3-fold symmetry axis of these genotypes in the modeled *T* = 3 arrangement. The models also predict that residues 112 and 189 may be closer together in GII.3, compared to GII.4 and GII.6. Based on both the sequence alignment and this structural modeling, we attempted to stabilize these GII genotypes by introducing N112C/N189C mutations, hereafter referred to as DS1.

**Fig. 2:**
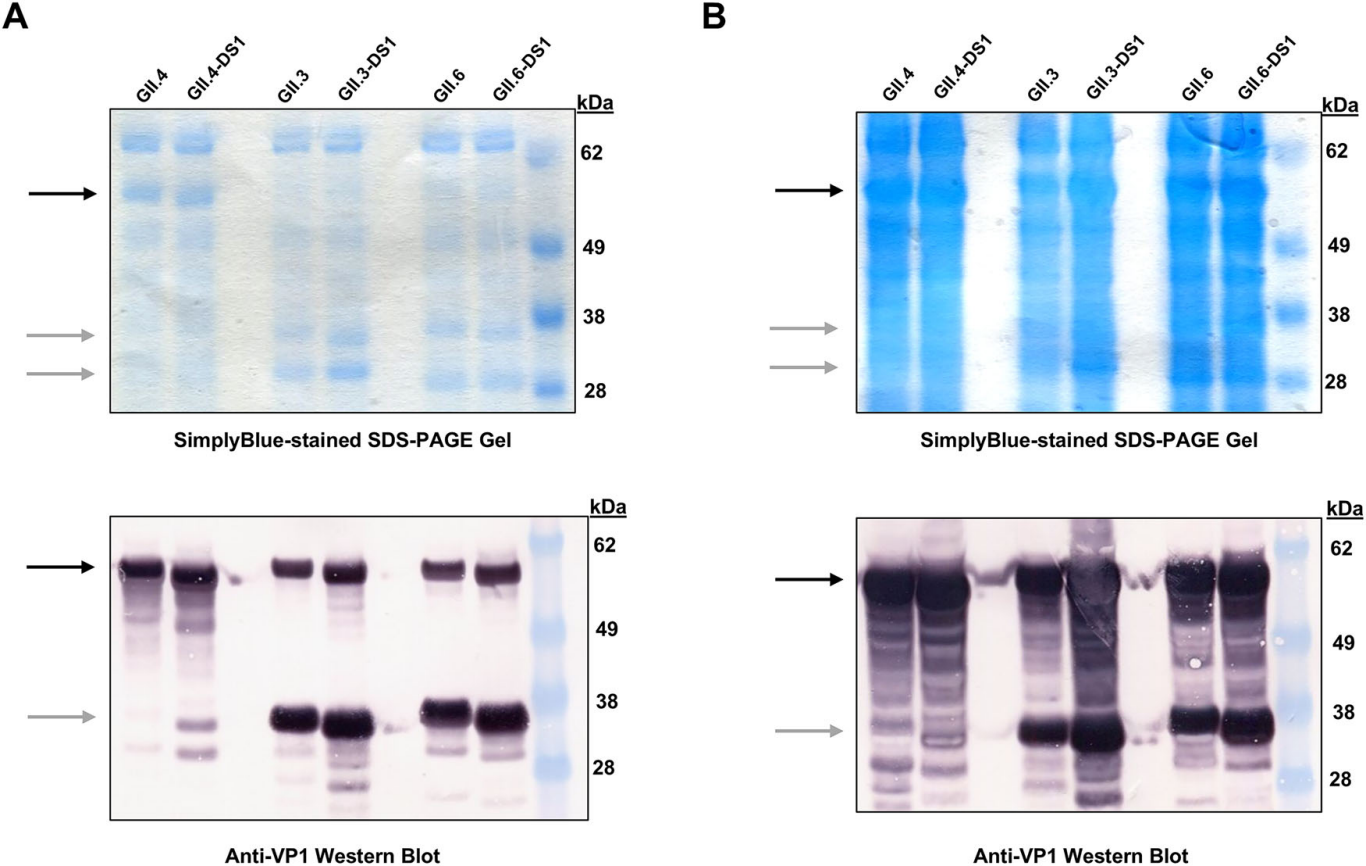
VP1 protein is expressed successfully from transiently transfected Expi293 cells. Cells were transfected with expression plasmids encoding various strains of norovirus VP1. Clarified supernatant (2.6 mg/lane, **A**) and lysate (15 mg/lane, **B**) fractions were separated via SDS-PAGE and either stained with SimplyBlue Safestain (upper panels) or blotted onto membranes and probed with an anti-VP1 antibody (lower panels). The position of full-length VP1 is denoted by black arrows. The positions of cleaved fragments of strains GII.3 and GII.6 are denoted by gray arrows. Marker proteins in kilodaltons (kDa) are indicated.

To determine VP1 protein expression, plasmids encoding VP1 protein from either GII.3, GII.4, GII.6 or their DS1 variants were transfected transiently into mammalian cells. In the supernatant, VP1 protein for GII.4 and GII.4-DS1 was readily visible by Coomassie-stained SDS-PAGE gel and anti-VP1 western blot whereas VP1 protein for GII.3 and GII.6 and their DS1 variants was primarily visible by anti-VP1 western blot only (Fig. 2A). In the cell lysate, VP1 protein is visible by both Coomassie-stained SDS-PAGE gel and anti-VP1 western blot for all constructs tested (Fig. 2B). Protease cleavage fragments are present for wildtype and DS1 variants for GII.3 and GII.6 in both the supernatant and cell lysate fractions (Fig. 2A, B), confirming earlier publications^36–40^. Purified GII.4 yielded an intact VLP of the expected size as determined by sucrose gradient, DLS and nsEM (Supplementary Fig. 1A, C, D). Additionally, purified GII.4 VLPs were thermostable as shown by nanoDSF (Supplementary Fig. 1B). Owing to the superior quality of GII.4 wildtype VLPs, we did not further evaluate GII.4-DS1 and focused our efforts on the DS1 variants of GII.3 and GII.6 VLPs.

### Engineered GII.3-DS1 and GII.6-DS1 assemble into VLPs and can form interprotomer disulfide bonds

**Fig. 3:**
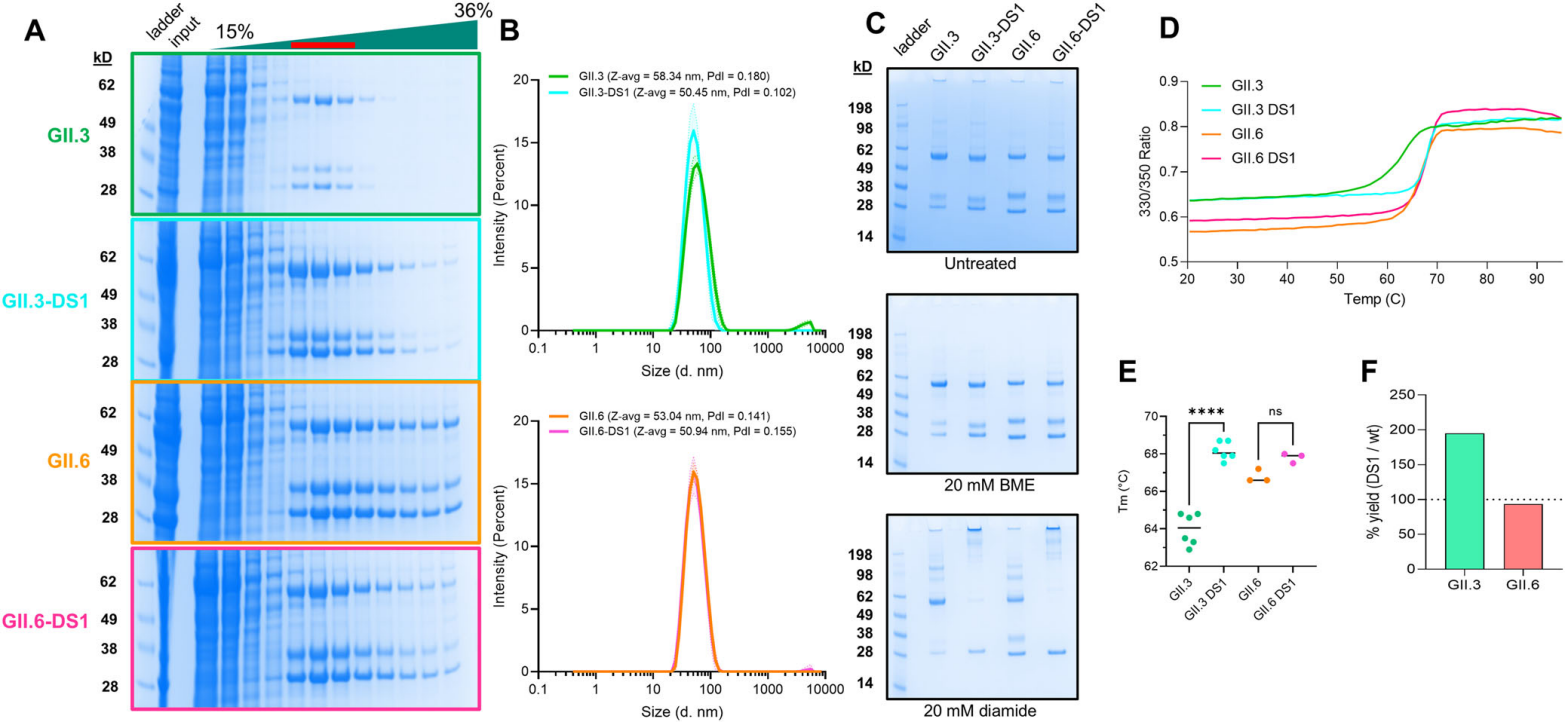
DS1 mutation stabilizes GII.3 VLPs. **A** Sucrose gradient purification of GII.3 and GII.6 VLPs with and without DS1 mutation. Fractions 5–7 (red line) were pooled for downstream analyses. Gradient input denoted input. **B** Dynamic light scattering (DLS) of GII.3 and GII.6 VLPs. Z-average (z-avg) and polydispersity index (PdI) values are shown in the insert. **C** SDS-PAGE analysis of GII.3 and GII.6 VLPs in either the untreated condition (top) or after treatment with 20 mM BME (middle) or 20 mM diamide (bottom) for 1 h at room temperature. **D** nanoDSF measurements of GII.3 and GII.6 VLPs. The line represents the average of 3 technical replicates. **E** Quantification of melting temperature (*T*_m_) from nanoDSF experiments, showing a significant increase in thermal stability of GII.3 VLPs upon introduction of the DS1 mutation. Significant values determined by unpaired *t*-test (*p* < 0.0001). **F** Relative yields of DS1 mutant compared to wild-type after sucrose gradient purification of VLPs.

Bulk harvested and clarified Expi293 cell lysates were analyzed via sucrose gradient ultracentrifugation to determine if the expressed GII.3 and GII.6 VP1 proteins and their DS1 counterparts form VLPs. Norovirus VLPs typically elute near the center of a 15–36% gradient (~25% sucrose), and we observed VLPs for all four samples (Fig. 3A). Lower molecular weight products of the protease-cleaved VP1 co-eluted with full-length VP1, indicating that these cleaved VP1 species are incorporated into VLPs. To determine if VP1 cleavage is required for VLP assembly and function, we also identified a conserved caspase cleavage site within the P-domains of GII.3 and GII.6 (Supplementary Fig. 2A–C). We show that mutations at this site in GII.3 (D302A/S) result in VLPs containing only full-length VP1 (Supplementary Fig. 2D). These VLPs have higher polydispersity and decreased saliva binding compared to wild-type GII.3 and GII.3-DS1 VLPs, consistent with previous studies (Supplementary Fig. 2F, G)^40^. Notably, compared to the GII.3 wild-type sequence, GII.3-DS1 showed much stronger bands in VLP fractions (Fig. 3A), indicating better expression and/or more efficient VLP formation. VLP fractions were pooled, dialyzed to remove sucrose, and concentrated to 0.5 mg/mL. Comparison of the relative post-sucrose gradient purification yields of GII.3 and GII.6 to their DS1 counterparts showed that indeed GII.3-DS1 generated approximately twice as much VLP as GII.3 while GII.6-DS1 and GII.6 yields were comparable (Fig. 3F).

To determine if disulfide bonds are formed in the DS1 mutants, VLPs were run on SDS-PAGE under 3 conditions: (1) without additional treatment, (2) after treatment with 20 mM beta-mercaptoethanol (reducing condition) for 1 h, or (3) after treatment with 20 mM diamide (oxidizing condition) for 1 h. We observed a distinct shift in the VP1 bands from GII.3-DS1 and GII.6-DS1 into the wells under oxidizing conditions (Fig. 3C). This shift is indicative of engineered disulfide bond formation. Disulfide bond formation was only observed on SDS-PAGE after treatment with a strong oxidizing agent; however, there may be a minor amount of VP1 material in the well in the untreated condition. This observation is consistent with previously published data on the GI.1-DS1 VLP^33^. All subsequent experiments were performed without diamide pretreatment.

### Disulfide stabilizing mutation enhances VLP formation, yield, and stability of strain GII.3 but not the closely related strain GII.6

To measure the effects of the DS1 mutation on VLP stability, we performed dynamic light scattering (DLS) and differential scanning fluorimetry (DSF) experiments. Compared to wild-type GII.3, GII.3-DS1 showed reductions in both the hydrodynamic diameter (58.34 to 50.45 nm) and polydispersity index (0.180 to 0.102), indicating GII.3-DS1 VLPs are more compact and more homogenous compared to wild-type GII.3 VLPs (Fig. 3B). The DS1 mutation did not appear to have a significant effect in the GII.6 background as measured by DLS. Compared to wild-type GII.6, GII.6-DS1 led to a slight reduction in the hydrodynamic diameter (53.04 to 50.94 nm) and a marginal increase in the polydispersity index (0.141 to 0.155) (Fig. 3B). We further conducted nanoDSF experiments to measure the thermal stability of VLPs. Wild-type GII.3 and GII.6 VLPs both showed monophasic transitions with melting temperatures(*T*_m_) of 64.0 and 66.8 °C, respectively (Fig. 3D, E). GII.3-DS1 showed a significant increase in *T*_m_ of 4.2 °C. In contrast, GII.6-DS1 marginally increased the *T*_m_ by 1.0 °C (Fig. 3D, E). Taken together, these data indicate that the DS1 mutation stabilizes GII.3 VLPs, whereas disulfide-induced stabilization was not observed for GII.6 VLPs.

**Fig. 4:**
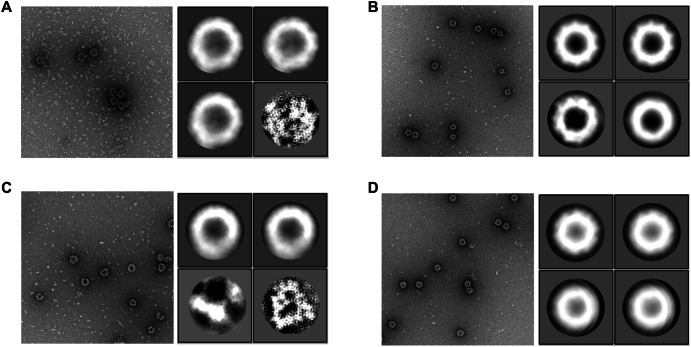
DS1 mutation leads to more uniform VLPs. Representative negative-stain electron micrographs (nsEM) and 2D classes of GII.3 (**A**), GII.3-DS1 (**B**), GII.6 (**C**), and GII.6-DS1 (**D**) VLPs. 2D class averages of intact VLPs are in the panel set on the right. nsEM magnification is 58,000X. VLPs were stored frozen at -80⁰C until processed for imaging. **A** GII.3 2D class averages derived from 139 particles picked from 27 micrographs. **B** GII.3-DS1 2D class averages derived from 97 particles picked from 26 micrographs. **C** GII.6 2D class averages derived from 205 particles picked from 27 micrographs. **D** GII.6-DS1 2D class averages derived from 314 particles picked from 24 micrographs.

To further assess VLP formation and stability, we imaged purified VLPs via negative-stain electron microscopy (nsEM). We directly compared sucrose gradient purified VLPs from genotypes GII.3, GII.3-DS1, GII.6 and GII.6-DS1 after a single freeze-thaw (Fig. 4) or without freezing (Supplementary Fig. 3). Intact VLPs were observed in all samples having diameters consistent with either *T* = 3 or *T* = 4 icosahedral symmetry, though we are unable to differentiate these two assembly types using nsEM. Micrographs from the 1x freeze thawed samples indicated a higher proportion of intact VLPs for GII.3-DS1 (Fig. 4B) compared to GII.3 (Fig. 4A), which showed a substantial amount of breakdown products in the micrographs. 2D classes generated from manually selected VLPs also confirmed more homogenous VLPs in the GII.3-DS1 sample compared to GII.3. This stabilizing effect was less pronounced when comparing wild-type GII.6 (Fig. 4C) and GII.6-DS1 (Fig. 4D) samples. The stabilizing effect was also less pronounced when comparing the VLPs without freeze-thaw (Supplementary Fig. 3), which showed fewer breakdown products in the wild-type GII.3 sample. Together, these biophysical and structural data indicate that the DS1 mutation stabilizes VLPs from the GII.3 genotype but has little effect on the closely related GII.6 genotype.

### GII.3 and GII.3-DS1 VLPs bind HBGA glycans and strain-specific monoclonal antibodies similarly

**Fig. 5:**
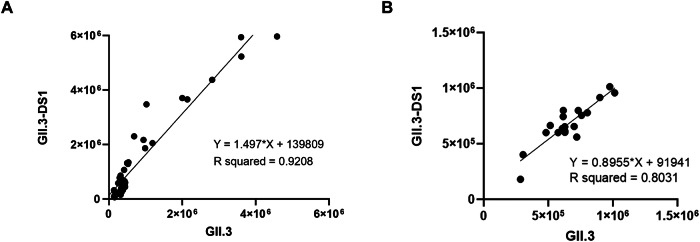
GII.3 and GII.3-DS1 VLPs bind to human saliva samples and monoclonal antibodies. **A** ELISA plates were coated with 51 human saliva samples and incubated with GII.3 or GII.3-DS1 VLPs at 20 µg/mL. Bound VLPs were detected using polyclonal sera against GII.3. Relative luminescent units (RLU) of GII.3 and GII.3-DS1 are reported on the *x-* and *y*-axes, respectively. Data are fitted into a simple linear regression model using GraphPad Prism software. **B** Plates were coated with 50 ng of either GII.3 or GII.3-DS1 VLP and incubated with 19 unique human monoclonal antibodies starting at 4 µg/mL and diluted in a four-fold, ten-point dilution series. Bound antibodies were detected using goat anti-human Ig-Fc fragment conjugated to horseradish peroxidase (HRP). Interpolated endpoint titer for each antibody against either GII.3 or GII.3-DS1 is reported on the *x*- and *y*-axes, respectively. Data are fitted into a simple linear regression model using GraphPad Prism software.

To assess the functionality of the GII.3 and GII.3-DS1 VLPs, we evaluated their ability to bind to histoblood group glycans present in human saliva. VLPs were used to bind a panel of human saliva samples on ELISA plates (*n* = 51). Results indicate that GII.3 and GII.3-DS1 VLPs bind to human saliva samples similarly (*R*^2^ = 0.9208) (Fig. 5A). Of note, consistent with previous observations, neither GII.3 nor GII.3-DS1 binds strongly to most salivary samples; however, we identified a few samples that showed high binding signals (Supplementary Fig. 4)^41,42^. The saliva that bound strongly to both GII.3 and GII.3-DS1 VLPs was used for all ELISA and HBGA assays hereafter. Furthermore, we screened a panel of GII.3-specific monoclonal antibodies (mAbs, *n* = 19) for their ability to bind GII.3 and GII.3-DS1 VLPs. We observed highly similar interpolated endpoint titers for both VLPs (*R*^2^ = 0.8031) (Fig. 5B). These similarities in the binding profiles indicate that GII.3-DS1 VLPs functionally mimic their wild-type counterpart.

### GII.3-DS1 mRNA elicited higher humoral responses in mice

**Fig. 6:**
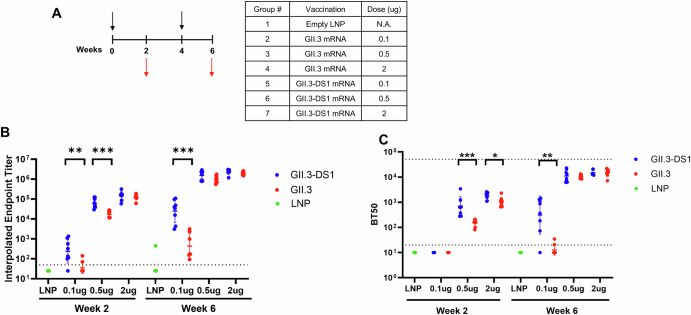
Mouse immunogenicity study of mRNAs encoding either GII.3 or GII.3-DS1, using assay reagent GII.3-DS1 VLP. **A** In vivo immunogenicity study design. BALB/c mice (*N* = 8 per group) were immunized with 0.1, 0.5, or 2 µg of mRNA at weeks 0 and 4 (black arrows). An empty lipid nanoparticle (LNP) was immunized as a negative control. Blood was drawn at weeks 2 and 6 (red arrows). Serum antibody ELISA titers (**B**) and HBGA blockade antibody titers (**C**) against GII.3-DS1 VLP. Serum of mice immunized with mRNAs encoding either GII.3 (red dots) or GII.3-DS1 (blue dots) was assayed. **B**, **C** Limits of detection are indicated with horizontal dotted lines (lower limit titer = 50 in (**B**) and 20 in (**C**), upper limit titer = 51,200 in (**C**). Geometric mean titers with geometric standard deviation are shown in a scatter dot plot. P values were determined using GraphPad Prism software (*p* < 0.05*, *p* < 0.01**, *p* < 0.001***).

We demonstrated that the DS1 mutation increases VLP quantities and confers increased stability to the GII.3 VLP without affecting glycan binding. To investigate whether the advantages conferred to the VLP by the DS1 mutation could improve the vaccine antigen, we vaccinated mice with mRNAs encoding GII.3 or GII.3-DS1 VP1 encapsulated in LNPs^43^. Mice were immunized twice, at four-week intervals, with three dose groups of each mRNA. Serum was collected at 2 weeks post doses 1 and 2 and evaluated for antibody binding and HBGA blockage titers (Fig. 6A). At both weeks 2 and 6, mRNA encoding GII.3-DS1 at low (0.1 µg) and mid (0.5 µg) doses elicited statistically significantly higher VLP binding ELISA titers than mRNAs encoding GII.3, although this difference was not observed at the high (2 µg) dose (Fig. 6B and Supplementary Table 1). While ELISA titers were detected in both low dose groups at week 2, neither GII.3 nor GII.3-DS1 mRNAs elicited measurable HBGA blocking titers at week 2 (Fig. 6C). However, at week 2, both the mid and high dose groups showed statistically significant differences, with GII.3-DS1 mRNA inducing higher HBGA blockage titers than GII.3 (Fig. 6C and Supplementary Table 1). By week 6, the low dose of GII.3-DS1 mRNAs elicited statistically significant HBGA blocking titers, while the mid and high doses of both GII.3-DS1 and GII.3 mRNAs performed similarly (Fig. 6C). Week 6 data remained consistent whether the assay regent used was GII.3 or GII-DS1 VLPs (Supplementary Fig. 5).

We next performed a series of mouse immunizations using purified VLPs to determine if the enhanced immunogenicity of GII.3-DS1 mRNA is due to increased VLP stability or if this effect can be more simply explained by higher expression of GII.3-DS1. Mice were immunized twice, at four-week intervals, with purified GII.3 or GII.3-DS1 VLPs at two doses: 0.2 and 2 µg. Immunizations were performed both with and without an aluminum salt (AAHS) adjuvant. In the high dose groups, we observed slight increases in both ELISA and HBGA blocking titers, both with and without adjuvant present (Supplementary Fig. 6A, C). However, in the low dose groups, we observed no statistically significant differences in the adjuvanted groups and even a modest decrease in immunogenicity of GII.3-DS1 VLPs in groups without AAHS adjuvant (Supplementary Fig. 6B, D). Despite these inconsistencies, the small magnitude of these differences from wild-type GII.3 VLPs strongly suggests that the robust increase in immunogenicity observed from the GII.3-DS1 mRNA can be largely attributed to increased expression of the antigen and not greater VLP stability in vivo. Taken together, these data suggest that, as an mRNA vaccine candidate, GII.3-DS1 is more immunogenic than the wild-type GII.3 at non-saturating dose levels in mice.

## Discussion

VLPs represent an attractive vaccine platform due to their structural mimicry of native virions and multivalent display of viral epitopes. Currently, recombinantly produced VLPs have been successfully implemented in two FDA-approved vaccines: Gardasil (HPV) and Engerix-B (HBV)^44^. Multiple enveloped and non-enveloped viruses may be amendable to future VLP-based vaccine development^45,46^. Independent of the vaccine modality, recombinant proteins are typically required for immunological assays to support vaccine development. In the case of norovirus, VLPs have been shown as promising vaccine candidates^47^. High-quality VLPs are required to support ELISA and HBGA functional assays, even if the vaccine platform is mRNA or adenovirus-based^25,48,49^. The manufacturing challenges posed by the inherent differences of VLPs across genotypes present an opportunity for the application of rational VLP engineering.

Here we demonstrate that a rationally engineered disulfide mutation (DS1) can significantly stabilize VLPs of the GII.3 genotype, leading to increased VLP yield and a significantly more thermostable product compared to wild-type. nsEM analysis further demonstrates the increased stability of GII.3-DS1 VLPs through the stress of a single freeze-thaw cycle. Our results are consistent with an earlier publication of the DS1 mutation in the archetypal GI.1 genotype, which showed increased thermostability of VLPs. These VLPs, in the absence of an adjuvant, also yielded superior HBGA blocking titers in an animal study^33^. We note that these GI.1-DS1 VLPs were treated with a strong oxidizing agent (diamide) during their purification, and all subsequent biophysical, structural, and immunological experiments were completed using the diamide-treated VLPs. In contrast, the GII.3-DS1 VLPs generated here show significantly enhanced stability without diamide treatment (Figs. 3 and 4). Consistent with this observation, we also demonstrated superior immune responses in a mouse immunogenicity study using mRNAs encoding GII.3-DS1 compared to wild-type GII.3 (Fig. 6). Due to the increased biophysical stability of untreated GII.3-DS1 VLPs, it was unexpected that a strong disulfide-induced band shift in SDS-PAGE was not observed unless VLPs were treated with diamide (Fig. 3C). We hypothesize that, in the untreated condition, GII.3-DS1 VLPs may transiently form disulfide bonds. In a *T* = 3 icosahedron with 180 copies of VP1, the DS1 mutation can form intermolecular disulfides at both the 5-fold and 3-fold symmetry axes, leading to a maximum of 180 possible intermolecular disulfide bonds formed per VLP. We theorize that even a small fraction of disulfides formed, while not detected by SDS-PAGE analysis, could increase overall VLP stability.

The advantages provided by the DS1 mutation are genotype-specific. Unlike GII.3, we did not observe a significant stabilizing effect of the DS1 mutation when introduced into the closely related GII.6 genotype, although GII.6-DS1 also showed disulfide bond formation after treatment with diamide (Fig. 3C). The reason for this difference is unclear; however, wild-type GII.6 VLPs were significantly more compact and less polydisperse than wild-type GII.3 by DLS (Fig. 3B) and showed higher thermal stability compared to wild-type GII.3 by nanoDSF (Fig. 3D, E). In addition, our structural modeling of the GII.6 shell in the *T* = 3 arrangement indicates that residues 112 and 189 may be less optimally positioned for disulfide bond engineering compared to GII.3 (Fig. 1E). Recently published cryoEM structures of multiple norovirus VLPs show significant variations in both the conformations of the shell domain and copy number, with the GII.4 Minerva strain predominantly existing as a *T* = 4 icosahedron containing 240 copies of VP1^21^. While this form represents a 33% increase in the number of VP1 protomers per VLP (240 vs. 180), the *T* = 4 VLPs are only approximately 16% larger in diameter compared to *T* = 3 VLPs from other GII genotypes (490 Å compared to 420 Å, respectively). This modest increase in diameter may not be detected by the DLS or nsEM methods employed here. Additionally, the DS1 mutation positions, N112C and N189C, are less optimal for stabilizing a *T* = 4 VLP, based on the GII.4 Minerva structure, compared to a *T* = 3 VLP^21^. Finally, multiple studies have demonstrated the existence of *T* = 1 VLPs, containing only 60 copies of VP1^21,50,51^. Among these studies, the GII.4 S_60_ nanoparticle was further stabilized by introducing a triple cysteine mutation within the shell domain (V57C/Q58C/S136C), which occurs at the two-fold symmetry axis and may be able to stabilize VLPs in multiple different arrangements. A similar mutation in GII.3 (Q58C/A136C) was screened in the current study but did not lead to significant increases in VP1 expression and therefore was not included in downstream analyses (data not shown). Taken together, we conclude that a universal approach to stabilize norovirus VLPs across genotypes is unlikely to be successful. Instead, precise modeling of local structure, VP1 dynamics and overall VLP arrangement would yield superior rationally designed stabilizing mutations for other genotypes.

We established that the DS1 mutation can stabilize GII.3 VLPs used as assay reagents. We sought to determine whether this stabilizing effect could be applied to improve GII.3 vaccine antigen design. In mice, mRNA encoding for GII.3-DS1 elicited significantly higher serum antibody titers compared to wild-type GII.3 mRNA (Fig. 6). We hypothesize that this result could be due to three possibilities: (1) increased VP1 protein production, (2) more efficient VLP formation, and/or (3) increased half-life of VLPs due to their enhanced stability. Immunizations with purified GII.3 and GII.3-DS1 VLPs show only very modest and inconsistent differences in ELISA and HBGA blocking titers among groups, suggesting that the increased immunogenicity of the GII.3-DS1 mRNA can be largely attributed to higher expression (Supplementary Fig. 6). Further supporting this hypothesis, we showed that GII.3-DS1 yielded greater amounts of VLP compared to GII.3 in transiently transfected Expi 293 cells (Fig. 3F). While it is unknown whether VLPs form within a mouse after vaccination with an mRNA-encoded VP1 sequence, previous structural studies of GII.3 show that the HBGA binding cleft is formed at the interprotomer interface of two P2 domains of assembled VP1 protein dimers, similar to HBGA binding sites in other GII strains^52,53^. Therefore, the presence of strong HBGA blocking titers, in addition to high ELISA titers, after mRNA vaccination is consistent with supramolecular assembly of VP1 proteins after mRNA vaccination.

Taken together, these data highlight the utility of structure-guided disulfide bond engineering for the stabilization of norovirus VLPs. These data also indicate that a universal approach to stabilize VLPs from different norovirus strains has a low probability of success due to intrinsic variations in VLP assembly and VP1 structure and dynamics. Finally, we demonstrate that the stabilizing mutations within GII.3-DS1 VLPs can be applied as an mRNA vaccine antigen, emphasizing the potential of rational vaccine design and development.

## Methods

### Structural modeling of *T* = 3 GII.3, GII.4, and GII.6 VP1 shell domains

Monomeric models of the shell domains from VP1 proteins (residues 1–220) were generated with AlphaFold2 using sequences for GII.3 (Genbank Accession MT409884), GII.4 (Genbank Accession MK754446), and GII.6 (Genbank Accession MH260487)^34^. High-confidence predictions (pLDDT >85) were generated for each shell sequence and three copies were subsequently aligned to the asymmetric unit of the GI.1-DS1 *T* = 3 shell structure (PDB 7kjp). These *T* = 3 models were subjected to energy minimization and FastRelax protocols in PyRosetta to remove any clashes that may have been generated during the structure alignment^35^. Intermolecular distances were measured between all Cα atoms from 2 loops containing residues 105–120 and 181–196 in the modeled *T* = 3 arrangements. 2D matrices corresponding to these intermolecular distances were plotted as a heatmap for visualization.

### Plasmid construction and VLP expression

Protein sequences of norovirus VP1 from genotypes GII.3 (Genbank Accession MT409884), GII.6 (Genbank Accession MH260487) and GII.4 (Genbank Accession MK754446) were mammalian codon-optimized by Genewiz (South Plainfield, NJ) and subcloned into a eukaryotic-expression vector under the control of the CMV promoter. DS1 variants were generated by introducing cysteine substitutions at amino acid positions N112 and N189 within the VP1 protein of each genotype. Caspase cleavage mutants (GII.3-D302A and GII.3-D302S) were generated by introducing alanine or serine substitutions at amino acid position D302 within VP1 of GII.3. Plasmids were transiently transfected into Expi 293 F cells (ThermoFisher, Waltham, MA) using Expifectamine (ThermoFisher, Waltham, MA) following the manufacturer’s recommended protocol. At 72 h post-transfection, a 1 mL sample of each culture was harvested into a microcentrifuge tube and centrifuged at 10,000 × *g*. Clarified supernatant was decanted into a fresh tube. The cell pellet was solubilized in 0.5 mL lysis buffer, centrifuged at 10,000 × *g*, and the clarified cell lysate was decanted into a fresh tube. Cell lysis buffer contained 0.5% Triton X-100, 40 mM Tris-HCl, pH 8.0, 120 mM NaCl, and 1X EDTA-free protease inhibitor (Roche Diagnostics, Indianapolis, IN). Clarified supernatant and cell lysate were used for SDS-PAGE and western blot analysis. Remaining bulk cell cultures were harvested 72 h post-transfection and frozen at −70 °C. To generate clarified bulk supernatant, the bulk harvests were thawed at room temperature (RT), mixed thoroughly by inversion, and centrifuged at 3500 × *g* for 10 min at RT. The resulting clarified bulk supernatant was transferred to a clean, sterile tube and stored at −70 °C.

### VLP purification

The frozen bulk cell culture was thawed and clarified through a Sartopure GF+ (Sartorius, Bohemia, NY) depth filter. The clarified VLPs were precipitated using 1.5 M ammonium sulfate (AS) and pelleted by centrifugation at 5000 × *g* for 10 min. The pellet was resuspended in an anion exchange chromatography (AEX) loading buffer and filtered through a 0.2 µm PVDF membrane (MilliporeSigma, Burlington, MA). The filtered AS precipitation product was purified by AEX using a Sartobind Q membrane (Sartorius, Bohemia, NY) and the VLPs were eluted in an elevated ionic strength buffer. The AEX eluates were further purified by size exclusion chromatography (SEC) using a Sepharose 6 FF (Cytiva, Marlborough, MA) column. Selected SEC fractions were pooled, concentrated, and exchanged into a formulation buffer using a 300 kDa UF/DF membrane (Repligen, Waltham, MA). The UF/DF product was spiked with a cryoprotectant and filtered through a 0.2 µm PVDF membrane (MilliporeSigma, Burlington, MA). All purified VLPs were frozen and stored at −70 °C. The final concentration of the VLPs was 1 mg/mL as measured by the Bradford Assay (ThermoFisher, Waltham, MA) using the manufacturer’s recommended protocol.

### SDS-PAGE and western blot

Clarified cell lysate and clarified supernatant were resolved on a 4–12% gradient, 1.5 mm, 15-well Bis-Tris NuPAGE gel in 1X MES buffer (ThermoFisher, Waltham, MA). For SDS-PAGE, where equal protein was loaded, the protein concentration was determined by Bradford assay. SeeBlue Plus2 pre-stained protein standard (ThermoFisher, Waltham, MA) was included on the SDS-PAGE for molecular weight reference. For Coomassie staining, SDS-PAGE gels were rinsed with water, stained with SimplyBlue Safestain (ThermoFisher, Waltham, MA) for 1 h at RT and destained with water at RT overnight. For Western blot analysis, proteins were transferred onto nitrocellulose membranes using the iBlot western blotting system (ThermoFisher, Waltham, MA). Membranes were transferred to the Bandmate Automated Western Blot Processor (ThermoFisher, Waltham, MA) for blocking and probing. Membranes were blocked for 1 h at RT with 30 mL of 5% non-fat dry milk (Blotting-grade blocker, Bio-Rad, Hercules, CA) in 1X tris-buffered saline containing 0.1% Tween-20 (1X TBST). Membranes were probed with a primary antibody cocktail of 2 mouse norovirus VP1 monoclonal antibodies at a dilution of 1:1000 in 5% non-fat dry milk in 1X TBST for 1 h at RT. The primary antibody cocktail consisted of a 1:1 mixture of two mouse norovirus VP1 monoclonal antibodies: My BioSource.com clone # M120539 (MyBioSource.com, San Diego, CA, cat # MBS832466) and Abcam clone # NVGC-01 (Abcam, Waltham, MA, cat # ab272687). Membranes were probed with an alkaline-phosphatase-conjugated affinipure goat anti-mouse IgG secondary antibody (Jackson ImmunoResearch, West Grove, PA, cat # 1115-055-146) diluted 1:1000 in 5% non-fat dry milk in 1X TBST for 1 h at RT. Membranes were washed 3 times for 5 min each at RT with 1X TBST. Membranes were developed using 1-step NBT/BCIP (Pierce, ThermoFisher, Waltham, MA) according to the manufacturer’s recommendations. Coomassie-stained SDS-PAGE gels and western blots were imaged using an Epson Perfection V370 Photo Scanner and Epson Scan Software version 3.9.2.5US.

### Sucrose gradient purification

30 mL frozen bulk lysates were thawed in a water bath, clarified by centrifugation, and concentrated to 1 mL using an Amicon 100 kDa MWCO centrifugal filter unit (MilliporeSigma, Burlington, MA). 5 μL of input material was saved for SDS-PAGE analysis. 16 mL, 15–36% continuous sucrose gradients in phosphate-buffered saline (PBS) were made on the day of purification using a Hoefer SG15 gradient maker (Hoefer, Bridgewater, MA) connected to a Cole Palmer Master Flex L/S Digital peristaltic pump flowed at 3.6 mL/min using size 16 tubing capped with a blunt 4-inch 14-gauge needle placed at the bottom of a 17 mL ultracentrifuge tube. 1 mL of concentrated bulk lysate was gently layered on top, and gradients were placed in SW28.1 buckets and loaded into a SW28Ti swinging bucket rotor (Beckman Coulter, Indianapolis, IN). Samples were centrifuged for 3 h at 150,000 × *g* in a Beckman Optima CL-100K Ultracentrifuge (Beckman Coulter, Indianapolis, IN) operated at 4 °C under vacuum. Gradients were manually fractionated from the top down using a pipette into 12 × 1.34 mL fractions. 15 μL of each fraction was analyzed on a 4–12% SDS-PAGE gel, and norovirus VLPs consistently eluted at the center of the gradient. Fractions 5–7 were pooled and dialyzed overnight using a 25 kDa MWCO dialysis cassette against norovirus VLP storage buffer containing: 25 mM Tris pH 7.5, 150 mM NaCl, 0.02% PS-80, and 5% sucrose. Protein concentration was estimated by Bradford assay using a BSA standard curve, and concentrations were adjusted to approximately 0.5 mg/mL for use in subsequent assays. VLPs were aliquoted, flash frozen in liquid nitrogen and stored at –80 °C.

### Dynamic light scattering (DLS)

The hydrodynamic diameter and polydispersity index (PdI) of purified VLPs were measured using a Malvern Zetasizer Nano ZS instrument (Malvern Panalytical, Westborough, MA). Measurements were recorded using 0.1 mL of purified VLPs at 0.5 mg/mL in norovirus VLP storage buffer (25 mM Tris, pH 7.5, 150 mM NaCl, 0.02% PS-80, and 5% (w/v) sucrose) in a ZEN040 cuvette (Malvern Panalytical, Westborough, MA) using the following instrument parameters:

- Material = Protein (RI = 1.450, Absorption = 0.001)
- Dispersant = 25 mM Tris, 150 mM NaCl, 5% (w/v) sucrose (Viscosity = 1.0454 cP, RI = 1.339)
- Temperature = 25 °C
- Method = Mark-Houwink
- Cell type = ZEN0040
- Equilibration time = 60 s
- Back scatter = 173⁰
- 3 measurements each (automatic duration)

General-purpose data analysis was performed in Zetasizer software. Intensity distribution values of the 3 replicates were plotted in GraphPad Prism software for visualization.

### VLP treatment

100 mM stocks of diamide (Sigma) and beta-mercaptoethanol (Acros Organics) were freshly prepared in PBS buffer. 2.5 µg of VLPs from each genotype were treated with either diamide or BME at a final concentration of 20 mM at RT in PBS buffer for 1 h. Samples were mixed with 1X LDS buffer (ThermoFisher, Waltham, MA) and boiled for 10 min prior to analysis via SDS-PAGE as described above.

### Nano differential scanning fluorimetry (nanoDSF)

Thermal stability assays were performed using a NanoTemper Prometheus NT.48 nanoDSF instrument (Nanotemper, Watertown, MA). Purified VLPs at a concentration of 0.5 mg/mL in norovirus VLP storage buffer were loaded into 3 NanoTemper high-sensitivity capillaries (3 technical replicates each). An initial discovery scan was used to optimize excitation power at 280 nm. Temperature was ramped from 20 °C to 95 °C at a rate of 1 °C per minute, and fluorescence was recorded at 330 nm and 350 nm. The 330/350 fluorescence ratio and its derivative were used to fit the inflection point (*T*_m_). The average 330/350 fluorescence signal of the 3 replicates was plotted in GraphPad Prism software for visualization.

### Electron microscopy

Purified VLP samples, along with norovirus VLP storage buffer, were shipped to Nano Imaging Services (NIS, San Diego, CA) for negative-stain electron microscopy analysis. The samples were diluted to a final concentration of 0.012 mg/mL with buffer. Samples were deposited on a layer of continuous carbon supported by nitrocellulose on a 400-mesh copper grid. The grids were prepared by applying 3 μL of sample suspension to a cleaned grid, blotting away with filter paper, and immediately staining with uranyl formate. Samples were imaged on an FEI Tecnai T12 electron microscope (serial number D1100), operating at 120 keV and equipped with an FEI Eagle 4k x 4k CCD camera. Negative-stain grids were transferred into the electron microscope using an RT stage. Data collection is carried out using Leginon software, where high magnification images are acquired by selecting targets at a lower magnification^54,55^. Images of each grid were acquired at multiple scales to assess the overall distribution of the specimen. After identifying potentially suitable target areas for imaging at lower magnifications, high magnification images were acquired at nominal magnifications of 110,000x (0.099 nm/pixel), 52,000x (0.212 nm/pixel) and 21,000x (0.513 nm/pixel). The images were acquired at a nominal underfocus of −5.0 μm to −1.2 μm and electron doses of ~10–25 e-/Å2. Representative micrographs for each sample collected at 52,000x were selected for qualitative comparisons. All micrographs from the 52,000x magnification were imported into RELION (v4.0.1)^56^ and CTF corrected using CTFFIND (v4.1.14)^57^ VLPs were manually picked from micrographs and were extracted in a 300 ×300 pixel (636 × 636 Å) box and subjected to 2D classification using 4 classes and a particle diameter of 500 Å.

### Saliva binding affinity assay

Maxisorp 384-well assay plates (Thermo Fisher, Waltham, MA) were coated with 25 μL of human saliva (Precision for Medicine, Frederick, MD) diluted 1:500 in Dulbecco’s phosphate-buffered saline (DPBS). Plates were sealed, centrifuged at 1500 × *g* for 1 min and incubated overnight at 4 °C in a humidity chamber. A norovirus VLP masterblock was generated by diluting VLPs in 5% non-fat dry milk powder (NFDMP) in phosphate-buffered saline containing 0.1% tween-20 (PBS-T) to a starting concentration of 20 μg/mL, followed by a two-fold, ten-point serial dilution. The masterblock was sealed, centrifuged at 1500 × *g* for 1 min and stored overnight at 4 °C in a humidity chamber. The assay plates were washed 6x with 1X PBS-T (100 µL/well) for 1 min each and blocked with 5% NFDMP in PBS-T (80 μL) for 30 min in a humidified incubator (21 °C, 80% RH). VLPs were added to 384-well assay plates and incubated for 1 h in a humidified incubator to allow for VLP binding of human saliva (21 °C, 80% RH). Following incubation, the assay plates were washed 6x with 1X PBS-T (100 µL/well) for 1 min each. Saliva-bound VLP was detected using GII.3-specific polyclonal rabbit serum in 5% NFDMP in PBS-T (1:3000) for 1 h in a humidified incubator (21 °C, 80% RH). Assay plates were washed 6x with PBS-T (100 μL/well) followed by the addition of goat anti-rabbit-HRP IgG (Jackson ImmunoResearch, West Grove, PA) diluted to 1:5000 in 5% NFDMP in PBS-T and incubated for 1 h in a humidified incubator (21 °C, 80% RH). After incubation, assay plates were washed 6x with PBS-T (100 μL/well). Luminescence was developed using 20 μL/well of luminescent substrate (Pierce West Pico PLUS, ThermoFisher, Waltham, MA) for 15 min at RT Ultrasensitive luminescence was read on an Envision plate reader at 0.1 s per well.

### HBGA blocking assay

Maxisorp 384-well assay plates (Thermo Fisher, Waltham, MA) were coated with human saliva (Precision for Medicine, Frederick, MD) diluted 1:500 in Dulbecco’s phosphate-buffered saline (DPBS). Assay plates were centrifuged at 1500 × *g* for 1 min and incubated overnight at 4 °C in a humidity chamber. Ten, two-fold serial dilutions of mouse sera were prepared in 5% non-fat dry milk powder in phosphate-buffered saline with Tween-20 (NFDMP-PBS-T) in 384-well masterblocks (Greiner Bio-One, Monroe, NC) with a starting dilution of 1:10. VLPs prepared at 1 µg/mL in 5% NFDMP-PBS-T were added in a 1:1 dilution to the mouse sera serial dilutions. Masterblocks were sealed, centrifuged at 1500 × *g* for 1 min and stored overnight at 4 °C in a humidity chamber. Assay plates were rinsed 6x with PBS-T and blocked with 5% NFDMP in PBS-T for 30 min in a humidified incubator (21 °C, 80% RH). VLP-mouse sera mixtures were transferred to assay plates and incubated for 2 h in a humidified incubator (21 °C, 80% RH). Assay plates were washed 6x with PBS-T. VLP was detected by incubation with GII.3-specific polyclonal rabbit serum diluted with 5% NFDMP in PBS-T (1:3000) for 1 h in a humidified incubator (21 °C, 80% RH). Assay plates were washed 6x with PBS-T. Goat anti-rabbit-HRP (Fc)-IgG (1:5000 in 5% NFDMP in PBS-T) (Jackson ImmunoResearch, West Grove, PA) was added to assay plates and incubated for 1 h in a humidified incubator (21 °C, 80% RH). Assay plates were washed 6x with PBS-T. Luminescent signal was developed for 15 min at RT with luminescent substrate (Pierce West Pico PLUS, ThermoFisher, Waltham, MA). BT50 values were defined as the titer at which luminescence readings were 50% of the positive control. A value of 10 was assigned to samples with a BT50 less than the starting dilution of 20. For all experiments, an anti-GII.3 mouse serum sample was included as a blocking control, and plates were rejected if the BT50 for the blocking control was greater than one dilution above or below the known BT50.

### Serum IgG and human GII.3 monoclonal antibody ELISA binding assays

Maxisorp 384-well plates (Thermo Fisher, Waltham, MA) were coated with 50 ng/well of VLP diluted in Dulbecco’s phosphate-buffered saline (DPBS). Plates were sealed, centrifuged at 1500 × *g* for 1 min and incubated overnight at 4 °C in a humidity chamber. Human IgG1 monoclonal antibodies, discovered internally by panning against a human Fab phage display library with norovirus VLPs as antigens, or mouse study serum samples, were diluted in DPBS to a starting concentration of 200 μg/mL (antibody) or 1:50 (serum) and subsequently diluted in masterblocks in a four-fold, ten-point series in 3% non-fat dry milk powder (NFDMP) in PBS containing 0.1% tween-20 (PBS-T). Dilution masterblocks were sealed, centrifuged at 1500 × *g* for 1 min and stored overnight at 4 °C in a humidity chamber. Assay plates were washed 6x with 1X PBS-T (100 μL/well) and blocked with 3% NFDMP in PBS-T (80 μL) for 30 min in a humidified incubator (21 °C, 80% RH). Monoclonal antibodies or serum were added to 384-well assay plates and incubated for 2 h in a humidified incubator to allow for VLP binding (21 °C, 80% RH). Following incubation, the assay plates were washed 6x with PBS-T (100 μL/well). VLP-bound antibodies were detected with HRP-goat anti-human IgG, Fcγ (min X) (Jackson ImmunoResearch, West Grove, PA, Catalog #109-035-098) or goat anti-mouse IgG (Fc), HRP conjugated (ThermoFisher Catalog #A16084) in 3% NFDMP in PBS-T (1:10,000) for 1 h in a humidified incubator (21 °C, 80% RH). Assay plates were washed 6x with PBS-T (100 μL/well) and developed with luminescent substrate (Pierce West Pico PLUS, ThermoFisher, Waltham, MA) for 15 min at RT. Ultrasensitive luminescence was read on an Envision plate reader (PerkinElmer, Waltham, MA) at 0.1 s per well. Interpolated serum antibody endpoint titers were calculated as the highest dilution where the relative light unit (RLU) signal is above a 50,000 RLU cutoff. Samples where no dilution crossed the threshold were given a placeholder titer of “25”. For samples above the threshold at the highest dilution tested (1:13107200), the value 13107200 was used for the titer. Samples where a well value was the same as 50,000 RLU were given the interpolated titer value at that exact dilution. If a sample crossed the 50000 RLU threshold between two dilutions, the titer at the crossing point was calculated by connecting the nearest points above and below the threshold, solving for the dilution at which this line crossed the threshold.

### mRNA/LNP generation

VP1-expressing mRNAs were generated by Trilink Biotechnologies (San Diego, CA) with N1-methyl-pseudouridine triphosphate modification and clean-cap. LNP-encapsulating mRNA was prepared by a rapid precipitation process as previously described^58^. The lipid components of the LNP comprised an asymmetric ionizable amino lipid, 1,2-distearoyl-sn-glycero-3-phosphocholine (DSPC), cholesterol and poly(ethylene glycol)2000-dimyristoylglycerol (PEG2000-DMG) in a molar ratio of 58:30:10:2, respectively.

### Rabbit polyclonal sera generation

The Institutional Animal Care and Use Committee at Merck & Co., Inc., Rahway, NJ, USA, approved the rabbit studies. Three female New Zealand white rabbits (approx. four months old at receipt) from Jackson Labs (Bar Harbor, ME) were housed singly and randomized into two groups. Animals were inoculated three times (two weeks between doses) intramuscularly in the quadriceps bilaterally. Inoculations consisted of 50 μg of appropriate VLP and 225 μg of amorphous aluminum hydroxyphosphate sulfate (AAHS) in 250 μL total (remaining volume was phosphate-buffered saline), administered once in each quadriceps at each time point. Blood draws were performed at weeks 1, 3, 5, and 6 after the first inoculation. All blood draws were from the ear artery, and the final was a terminal bleed. For all survival blood draws, animals were dosed with acepromazine (1–3 mg/kg subcutaneously) and approximately 2 mL of blood was drawn from the ear artery. For terminal bleeds, animals were administered ketamine (100 mg) and xylazine (20 mg) intramuscularly, then were bled from the ear artery until no more blood could be acquired. A cardiac puncture was performed for the remaining blood, and then euthanasia was performed by administration of Euthasol intravenously. Euthanasia was confirmed by cutting the diaphragm to cause pneumothorax. All blood was spun at 4000 ×*g* for 5 min to separate serum, which was transferred to new tubes.

### Mouse immunization studies

The Institutional Animal Care and Use Committee at Merck & Co., Inc., Rahway, NJ, USA, approved the mouse studies. BALB/c mice aged 6–8 weeks were obtained from Charles River Laboratory (Malvern, PA). Animals were housed in a company animal facility, in accordance with the Guide for the Care and Use of Laboratory Animals, and the facility is credentialed by the Association for Assessment and Accreditation of Laboratory Animal Care^59^. Seven groups of mice (*N* = 8/group) were immunized at weeks 0 and 4 with intramuscular injections of mRNAs in a lipid nanoparticle formulation. Mice were injected with 100 μL total volume per vaccination with 50 μL injected per thigh. Two weeks after the first immunization, blood was drawn via the tail vein. Specifically, a small cut was made on the tail vein with a scalpel blade (Bard-Parker, Size 15, Fisher Catalog #0268880) and around 100–150 μL blood was collected into serum separator tubes (Sarstedt AG & Co. KG Microvette 500 Z-Gel Catalog #20.1344). Two weeks after the second immunization, all mice were euthanized via CO_2_ exposure. Blood collection was performed via cardiac puncture using a 1 mL syringe with a 25G needle (BD Catalog #309626). Blood was transferred into serum separator tubes. Euthanasia was confirmed by cervical dislocation. For VLP immunogen studies, nine groups of mice (*N* = 10 /group) were immunized with 2 μg or 0.2 μg of either GII.3 or GII.3-DS1 VLPs with and without amorphous aluminum hydroxyphosphate sulfate (AAHS) at weeks 0 and 4. Mice were injected intramuscularly with 100 μL total volume, 50 μL in each thigh. Blood was drawn at weeks 2, 6, and 10.

### Statistical analysis

Statistical analyses were completed using GraphPad Prism version 10.2.2. Data were tested for normality using a Shapiro–Wilk test. If the normality of both groups is satisfied, then statistical significance was determined by an unpaired two-sample *t*-test with Welch’s correction. If normality is not satisfied, statistical significance was determined using a Mann–Whitney *U*-test.

### Edman degradation sequencing

N-terminal Edman degradation sequencing was outsourced to Evotec (Princeton, NJ). Purified GII.3 VLPs were shipped at 4 °C to Evotec. The N-termini of the full-length VP1 protein and 28 kDa cleavage product were sequenced to determine the site of VP1 cleavage within the cell during expression.

## Supplementary information

8July2025Supp

## Data Availability

Data were plotted and visualized using GraphPad Prism version 10.2.2. Data generated and analyzed in the current study are included in this published article and its supplementary information files. All nucleotide sequences have been deposited in GenBank (https://www.ncbi.nlm.nih.gov/genbank/) under accession numbers PV921589-PV921598. nsEM datasets are available from the corresponding author upon request. Non-commercial critical reagents are available upon request. Readers may contact the corresponding author to request reagents or materials via a Material Transfer Agreement (MTA).

## Code availability

No custom scripts were generated in this study. Electron micrographs were analyzed using RELION (v4.0.1) for CTF correction, particle picking, and 2D classification.

## References

[1] Lopman, B. A., Steele, D., Kirkwood, C. D. & Parashar, U. D. The vast and varied global burden of norovirus: prospects for prevention and control. *PLoS Med.* **13**, e1001999 (2016). 27115709 10.1371/journal.pmed.1001999 PMC4846155

[2] Teunis, P. F. et al. Norwalk virus: how infectious is it?. *J. Med. Virol.* **80**, 1468–1476 (2008). 18551613 10.1002/jmv.21237

[3] de Graaf, M., van Beek, J. & Koopmans, M. P. Human norovirus transmission and evolution in a changing world. *Nat. Rev. Microbiol.* **14**, 421–433 (2016). 27211790 10.1038/nrmicro.2016.48

[4] Verhoef, L. et al. Norovirus genotype profiles associated with foodborne transmission, 1999–2012. *Emerg. Infect. Dis.* **21**, 592–599 (2015). 25811368 10.3201/eid2104.141073 PMC4378480

[5] Bartsch, S. M., Lopman, B. A., Ozawa, S., Hall, A. J. & Lee, B. Y. Global economic burden of norovirus gastroenteritis. *PLoS One* **11**, e0151219 (2016). 27115736 10.1371/journal.pone.0151219 PMC4846012

[6] Ahmed, S. M. et al. Global prevalence of norovirus in cases of gastroenteritis: a systematic review and meta-analysis. *Lancet Infect. Dis.* **14**, 725–730 (2014). 24981041 10.1016/S1473-3099(14)70767-4 PMC8006533

[7] U.S. Centers for Disease Control and Prevention. *Norovirus Facts and Stats* https://www.cdc.gov/norovirus/data-research/?CDC_AAref_Val=, https://www.cdc.gov/norovirus/burden.html (U.S. Centers for Disease Control and Prevention, 2024, May 8).

[8] Chhabra, P. et al. Corrigendum: Updated classification of norovirus genogroups and genotypes. *J. Gen. Virol.* **101**, 893 (2020). 32854814 10.1099/jgv.0.001475 PMC7641392

[9] Hall, A. J. et al. Norovirus disease in the United States. *Emerg. Infect. Dis.* **19**, 1198–1205 (2013). 23876403 10.3201/eid1908.130465 PMC3739528

[10] Wangchuk, S., Matsumoto, T., Iha, H. & Ahmed, K. Surveillance of norovirus among children with diarrhea in four major hospitals in Bhutan: replacement of GII.21 by GII.3 as a dominant genotype. *PLoS ONE* **12**, e0184826 (2017). 28910371 10.1371/journal.pone.0184826 PMC5599041

[11] Boonchan, M. et al. The dynamics of norovirus genotypes and genetic analysis of a novel recombinant GII.P12-GII.3 among infants and children in Bangkok, Thailand between 2014 and 2016. *Infect. Genet. Evol.* **60**, 133–139 (2018). 29471118 10.1016/j.meegid.2018.02.028

[12] Utsumi, T. et al. Molecular epidemiology and genetic diversity of norovirus infection in children hospitalized with acute gastroenteritis in East Java, Indonesia in 2015-2019. *Infect. Genet Evol.* **88**, 104703. 10.1016/j.meegid.2020.104703 (2021). 33401005 10.1016/j.meegid.2020.104703

[13] Cannon, J. L. et al. Global trends in norovirus genotype distribution among children with acute gastroenteritis. *Emerg. Infect. Dis.* **27**, 1438–1445 (2021). 33900173 10.3201/eid2705.204756 PMC8084493

[14] NoroSurv. *Norosurv.org*, https://www.norosurv.org/login (NoroSurv., 2025).

[15] Jiang, X., Wang, M., Graham, D. Y. & Estes, M. K. Expression, self-assembly, and antigenicity of the Norwalk virus capsid protein. *J. Virol.* **66**, 6527–6532 (1992). 1328679 10.1128/jvi.66.11.6527-6532.1992 PMC240146

[16] Prasad, B. V. et al. X-ray crystallographic structure of the Norwalk virus capsid. *Science* **286**, 287–290 (1999). 10514371 10.1126/science.286.5438.287

[17] Prasad, B. V., Rothnagel, R., Jiang, X. & Estes, M. K. Three-dimensional structure of baculovirus-expressed Norwalk virus capsids. *J. Virol.* **68**, 5117–5125 (1994). 8035511 10.1128/jvi.68.8.5117-5125.1994 PMC236455

[18] Bertolotti-Ciarlet, A., White, L. J., Chen, R., Prasad, B. V. & Estes, M. K. Structural requirements for the assembly of Norwalk virus-like particles. *J. Virol.* **76**, 4044–4055 (2002). 11907243 10.1128/JVI.76.8.4044-4055.2002 PMC136079

[19] Vongpunsawad, S., Venkataram Prasad, B. V. & Estes, M. K. Norwalk virus minor capsid protein VP2 associates within the VP1 shell domain. *J. Virol.* **87**, 4818–4825 (2013). 23408637 10.1128/JVI.03508-12 PMC3624303

[20] Bertolotti-Ciarlet, A., Crawford, S. E., Hutson, A. M. & Estes, M. K. The 3’ end of Norwalk virus mRNA contains determinants that regulate the expression and stability of the viral capsid protein VP1: a novel function for the VP2 protein. *J. Virol.* **77**, 11603–11615 (2003). 14557646 10.1128/JVI.77.21.11603-11615.2003 PMC229252

[21] Jung, J. et al. High-resolution cryo-EM structures of outbreak strain human norovirus shells reveal size variations. *Proc. Natl. Acad. Sci. USA* **116**, 12828–12832 (2019). 31182604 10.1073/pnas.1903562116 PMC6601263

[22] Choi, J. M., Hutson, A. M., Estes, M. K. & Prasad, B. V. Atomic resolution structural characterization of recognition of histo-blood group antigens by Norwalk virus. *Proc. Natl. Acad. Sci. USA* **105**, 9175–9180 (2008). 18599458 10.1073/pnas.0803275105 PMC2453692

[23] Treanor, J. J. et al. A novel intramuscular bivalent norovirus virus-like particle vaccine candidate-reactogenicity, safety, and immunogenicity in a phase 1 trial in healthy adults. *J. Infect. Dis.* **210**, 1763–1771 (2014). 24951828 10.1093/infdis/jiu337 PMC8483568

[24] Fang, H., Tan, M., Xia, M., Wang, L. & Jiang, X. Norovirus P particle efficiently elicits innate, humoral and cellular immunity. *PLoS One* **8**, e63269 (2013). 23638188 10.1371/journal.pone.0063269 PMC3639243

[25] Kim, L. et al. Safety and immunogenicity of an oral tablet norovirus vaccine, a phase I randomized, placebo-controlled trial. *JCI Insight* **3**10.1172/jci.insight.121077 (2018). 10.1172/jci.insight.121077 PMC6124525 29997294

[26] Atmar, R. L. et al. An exploratory study of the salivary immunoglobulin A responses to 1 dose of a norovirus virus-like particle candidate vaccine in healthy adults. *J. Infect. Dis.* **219**, 410–414 (2019). 30203081 10.1093/infdis/jiy529 PMC8483565

[27] Wrapp, D. et al. Cryo-EM structure of the 2019-nCoV Spike in the prefusion conformation. *bioRxiv*10.1101/2020.02.11.944462 (2020). 32075877 10.1126/science.abb2507 PMC7164637

[28] Pallesen, J. et al. Immunogenicity and structures of a rationally designed prefusion MERS-CoV spike antigen. *Proc. Natl. Acad. Sci. USA* **114**, E7348–E7357 (2017). 28807998 10.1073/pnas.1707304114 PMC5584442

[29] Hsieh, C. L. et al. Structure-based design of prefusion-stabilized SARS-CoV-2 spikes. *Science* **369**, 1501–1505 (2020). 32703906 10.1126/science.abd0826 PMC7402631

[30] Tan, T. J. C. et al. High-throughput identification of prefusion-stabilizing mutations in SARS-CoV-2 spike. *Nat. Commun.* **14**, 2003 (2023). 37037866 10.1038/s41467-023-37786-1 PMC10086000

[31] Papi, A. et al. Respiratory syncytial virus prefusion F protein vaccine in older adults. *N. Engl. J. Med.* **388**, 595–608 (2023). 36791160 10.1056/NEJMoa2209604

[32] McLellan, J. S. et al. Structure-based design of a fusion glycoprotein vaccine for respiratory syncytial virus. *Science* **342**, 592–598 (2013). 24179220 10.1126/science.1243283 PMC4461862

[33] Verardi, R. et al. Disulfide stabilization of human norovirus GI.1 virus-like particles focuses immune response toward blockade epitopes. *NPJ Vaccines* **5**, 110 (2020). 33318483 10.1038/s41541-020-00260-w PMC7736355

[34] Jumper, J. et al. Highly accurate protein structure prediction with AlphaFold. *Nature* **596**, 583–589 (2021). 34265844 10.1038/s41586-021-03819-2 PMC8371605

[35] Chaudhury, S., Lyskov, S. & Gray, J. J. PyRosetta: a script-based interface for implementing molecular modeling algorithms using Rosetta. *Bioinformatics* **26**, 689–691 (2010). 20061306 10.1093/bioinformatics/btq007 PMC2828115

[36] Ma, S. et al. Chimeric GII.3/GII.6 norovirus capsid (VP1) proteins: characterization by electron microscopy, trypsin sensitivity and binding to histo-blood group antigens. *Arch. Virol.* **163**, 3265–3273 (2018). 30143876 10.1007/s00705-018-4002-8

[37] Hardy, M. E., White, L. J., Ball, J. M. & Estes, M. K. Specific proteolytic cleavage of recombinant Norwalk virus capsid protein. *J. Virol.* **69**, 1693–1698 (1995). 7853506 10.1128/jvi.69.3.1693-1698.1995 PMC188770

[38] Kumar, S., Ochoa, W., Kobayashi, S. & Reddy, V. S. Presence of a surface-exposed loop facilitates trypsinization of particles of Sinsiro virus, a genogroup II.3 norovirus. *J. Virol.* **81**, 1119–1128 (2007). 17079293 10.1128/JVI.01909-06 PMC1797490

[39] Zheng, L. et al. Comprehensive characterization of a major capsid protein derived from a documented GII.6 norovirus strain. *Arch. Virol.* **162**, 3863–3868 (2017). 28866835 10.1007/s00705-017-3537-4

[40] Huo, Y. et al. Enzymatic cleavage promotes disassembly of GII.3 norovirus virus like particles and its binding to salivary histo-blood group antigens. *Virus Res*. **240**, 18–24 (2017). 28754559 10.1016/j.virusres.2017.07.017

[41] Huo, Y., Wan, X., Ling, T. & Shen, S. Biological and immunological characterization of norovirus major capsid proteins from three different genotypes. *Micro Pathog.* **90**, 78–83 (2016). 10.1016/j.micpath.2015.11.022 26616166

[42] Ayouni, S. et al. Relationship between GII.3 norovirus infections and blood group antigens in young children in Tunisia. *Clin. Microbiol Infect.* **21**, 874.e871–874.e878 (2015). 10.1016/j.cmi.2015.05.015 26003283

[43] Swaminathan, G. et al. A novel lipid nanoparticle adjuvant significantly enhances B cell and T cell responses to sub-unit vaccine antigens. *Vaccine* **34**, 110–119 (2016). 26555351 10.1016/j.vaccine.2015.10.132

[44] Kheirvari, M., Liu, H. & Tumban, E. Virus-like particle vaccines and platforms for vaccine development. *Viruses* **15**10.3390/v15051109 (2023). 10.3390/v15051109 PMC10223759 37243195

[45] Nooraei, S. et al. Virus-like particles: preparation, immunogenicity and their roles as nanovaccines and drug nanocarriers. *J. Nanobiotechnol.* **19**, 59 (2021). 10.1186/s12951-021-00806-7 PMC7905985 33632278

[46] Chaudhary, N., Weissman, D. & Whitehead, K. A. mRNA vaccines for infectious diseases: principles, delivery and clinical translation. *Nat. Rev. Drug Discov.* **20**, 817–838 (2021). 34433919 10.1038/s41573-021-00283-5 PMC8386155

[47] Leroux-Roels, G., Atmar, R. L., Cramer, J. P., Escudero, I. & Borkowski, A. Persistence of the immune response to an intramuscular bivalent (GI.1/GII.4) Norovirus vaccine in adults. *Vaccines* **13**10.3390/vaccines13010082 (2025). 10.3390/vaccines13010082 PMC11768790 39852862

[48] Bollman, B., Jorquera, P. & Arunkumar, G. Norovirus mRNA Vaccines. *World Intellectual Property Organization* WO2024015890 (2024).

[49] Schoofs, T. et al. 575. Safety and immunogenicity of mRNA-1403, a multivalent norovirus mRNA vaccine, in Healthy Adults: Interim Results of a Phase 1/2, Randomized, Observer-Blind, Placebo-Controlled, Dose-Ranging Trial. *Open Forum Infect. Dis.* **12**10.1093/ofid/ofae631.013 (2025).

[50] Xia, M. et al. Bioengineered norovirus S(60) nanoparticles as a multifunctional vaccine platform. *ACS Nano* **12**, 10665–10682 (2018). 30234973 10.1021/acsnano.8b02776 PMC6261714

[51] Devant, J. M. & Hansman, G. S. Structural heterogeneity of a human norovirus vaccine candidate. *Virology* **553**, 23–34 (2021). 33202318 10.1016/j.virol.2020.10.005

[52] Yang, Y. et al. Structural basis of host ligand specificity change of GII porcine noroviruses from their closely related GII human noroviruses. *Emerg. Microbes Infect.* **8**, 1642–1657 (2019). 31711377 10.1080/22221751.2019.1686335 PMC6853222

[53] Hansman, G. S. et al. Crystal structures of GII.10 and GII.12 norovirus protruding domains in complex with histo-blood group antigens reveal details for a potential site of vulnerability. *J. Virol.* **85**, 6687–6701 (2011). 21525337 10.1128/JVI.00246-11 PMC3126497

[54] Suloway, C. et al. Automated molecular microscopy: the new Leginon system. *J. Struct. Biol.* **151**, 41–60 (2005). 15890530 10.1016/j.jsb.2005.03.010

[55] Cheng, A. et al. Leginon: new features and applications. *Protein Sci.* **30**, 136–150 (2021). 33030237 10.1002/pro.3967 PMC7737759

[56] Scheres, S. H. RELION: implementation of a Bayesian approach to cryo-EM structure determination. *J Struct Biol* **180**, 519–530 (2012). 23000701 10.1016/j.jsb.2012.09.006 PMC3690530

[57] Rohou, A. & Grigorieff, N. CTFFIND4: Fast and accurate defocus estimation from electron micrographs. *J Struct Biol* **192**, 216–221 (2015). 26278980 10.1016/j.jsb.2015.08.008 PMC6760662

[58] Gindy, M. E. et al. Stabilization of Ostwald ripening in low molecular weight amino lipid nanoparticles for systemic delivery of siRNA therapeutics. *Mol. Pharm.* **11**, 4143–4153 (2014). 25317715 10.1021/mp500367k

[59] National Research Council Committee for the Update of the Guide for the Care and Use of Laboratory Animals. *The National Academies Collection: Reports funded by National Institutes of Health*. (National Academies Press (US), 2011).

