# Supplementary material for: Stabilization of norovirus GII.3 virus-like particles by rational disulfide engineering
Source: NPJ Vaccines. 2025 Aug 19;10:196. doi: 10.1038/s41541-025-01254-2 (PMC12365219; doi:10.1038/s41541-025-01254-2)
Supplement: Supplementary file 1 — 8July2025Supp [file 41541_2025_1254_MOESM1_ESM.pdf]

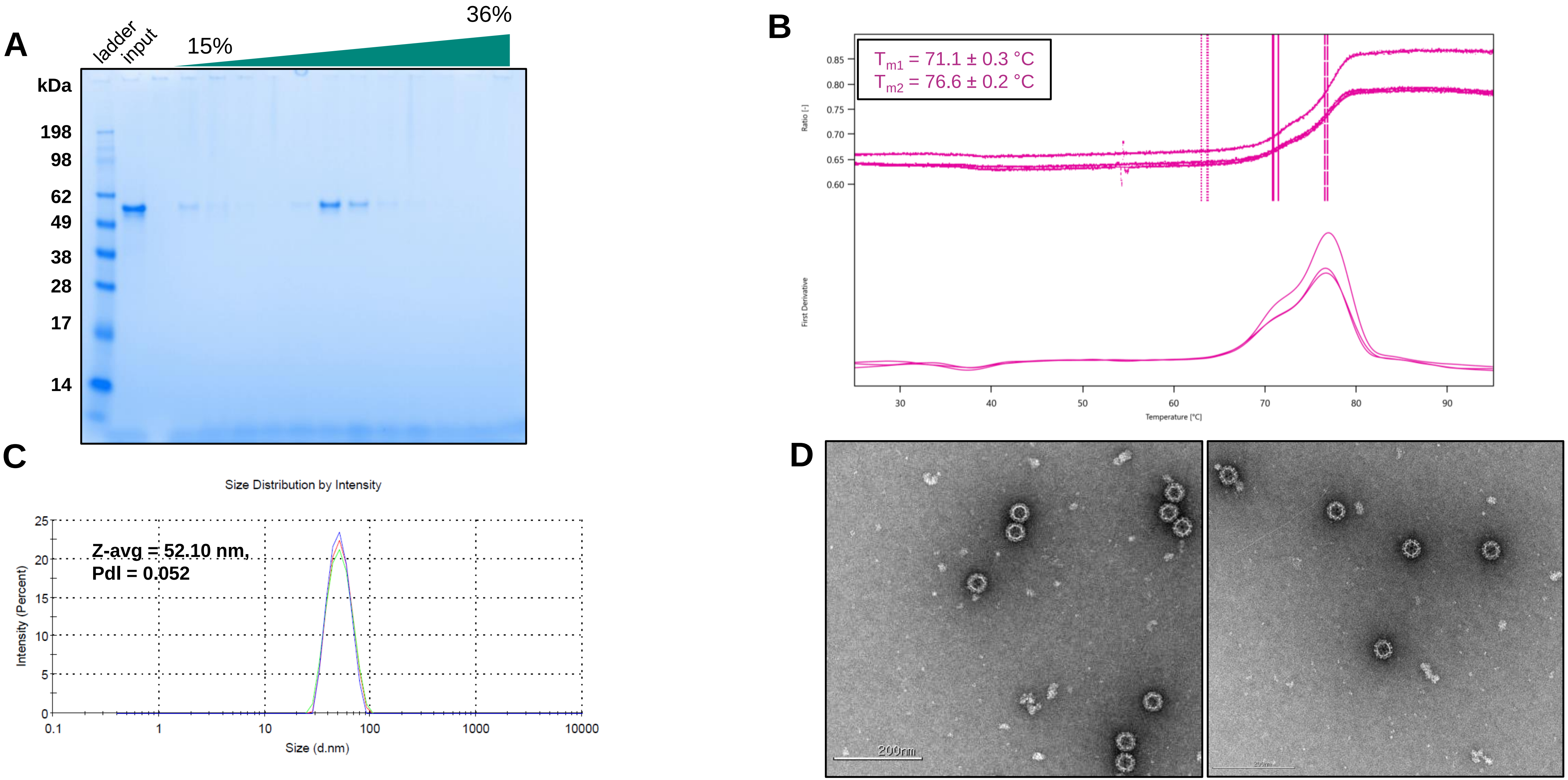

Supplemental Figure 1– GII.4 VLPs are stable without the DS1 mutation addition.
A Sucrose gradient fractionation of fully purified GII.4. VP1 protein observed in fractions 5-7 at ~ 25% sucrose, the percentage at which VLPs are known to fractionate. Gradient input denoted inp. **B** Differential scanning fluorimetry (DSF) was used to determine the thermal stability of GII.4 VLP. GII.4 VLP shows a biphasic transition with melting temperatures (T_m) of 71.1 °C and 76.6 °C. **C** Dynamic light scattering (DLS) of GII.4 VLP. Samples were run in triplicate. GII.4 VLP was determined to be homogeneous, compact, and monodisperse (polydispersity index of 0.052) with a hydrodynamic radius of 52.10 nm. **D** Representative negative-stain electron micrograph (nsEM) of GII.4 VLP. nsEM shows intact VLPs with few breakdown or partially assembled VP1 protomers.

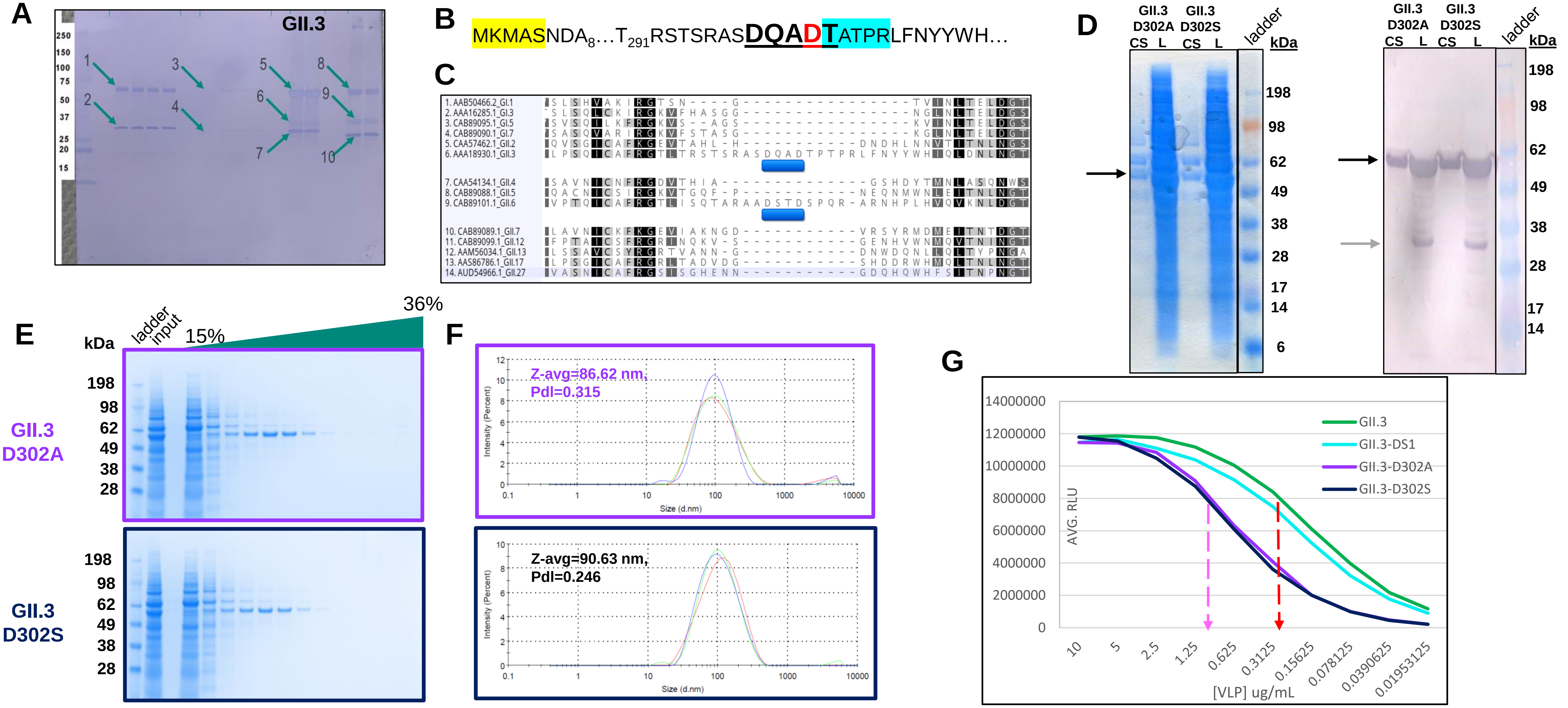

Supplemental Figure 2 - Reduction of GII.3 VP1 cleavage results in decreased glycan binding. A Coomassie-stained, reduced and denatured SDS-PAGE gel of GII.3 VLP. GII.3 VP1 proteins sequenced by N-terminal Edman degradation noted as bands 5-7. **B** GII.3 VP1 amino acid sequence of Edman sequenced region. N-terminus of protein band 5 highlighted in yellow. N-terminus of protein band 7 highlighted in cyan. Caspase cleavage consensus site in bold and underlined. Amino acid targeted for mutation in bold red font. **C** Alignment of the cleaved region in multiple circulating GI and GII genotype VP1 sequences. Blue marker indicates the caspase cleavage consensus site found in GII.3 and GII.6. **D** VP1 expression of GII.3-D302A and GII.3-D302S in Expi293 cells. Clarified supernatant (CS) and lysate (L) fractions were separated via SDS-PAGE and either stained with SimplyBlue Safestain (left panel) or blotted onto a membrane and probed with an anti-VP1 antibody (right panel). The position of full length VP1 is denoted by black arrows. The position of cleaved fragments is denoted by grey arrows. Marker proteins in kilodalton (kDa) are indicated. **E** Sucrose gradient purification of GII.3-D302A and GII.3-D302S VLP from clarified supernatant. Fractions 5-7 (red line) were pooled for downstream analyses. Gradient input denoted inp. **F** Dynamic light scattering (DLS) of GII.3-D302A and GII.3-D302S VLPs, run in triplicate. Z-average (z-avg) and polydispersity index (PdI) values shown in insert. **G** Binding of various GII.3 VLPs to human saliva from a single human donor. VLPs were diluted to 10 ug/ml and diluted in a 10 point, 2-fold serial dilution series. Binding curves were completed in quadruplicate and the average RLU for each point was plotted. Titration curve midpoint shown for GII.3/GII.3-DS1 (red) and GII.3-D302A/GII.3-D302S (pink).

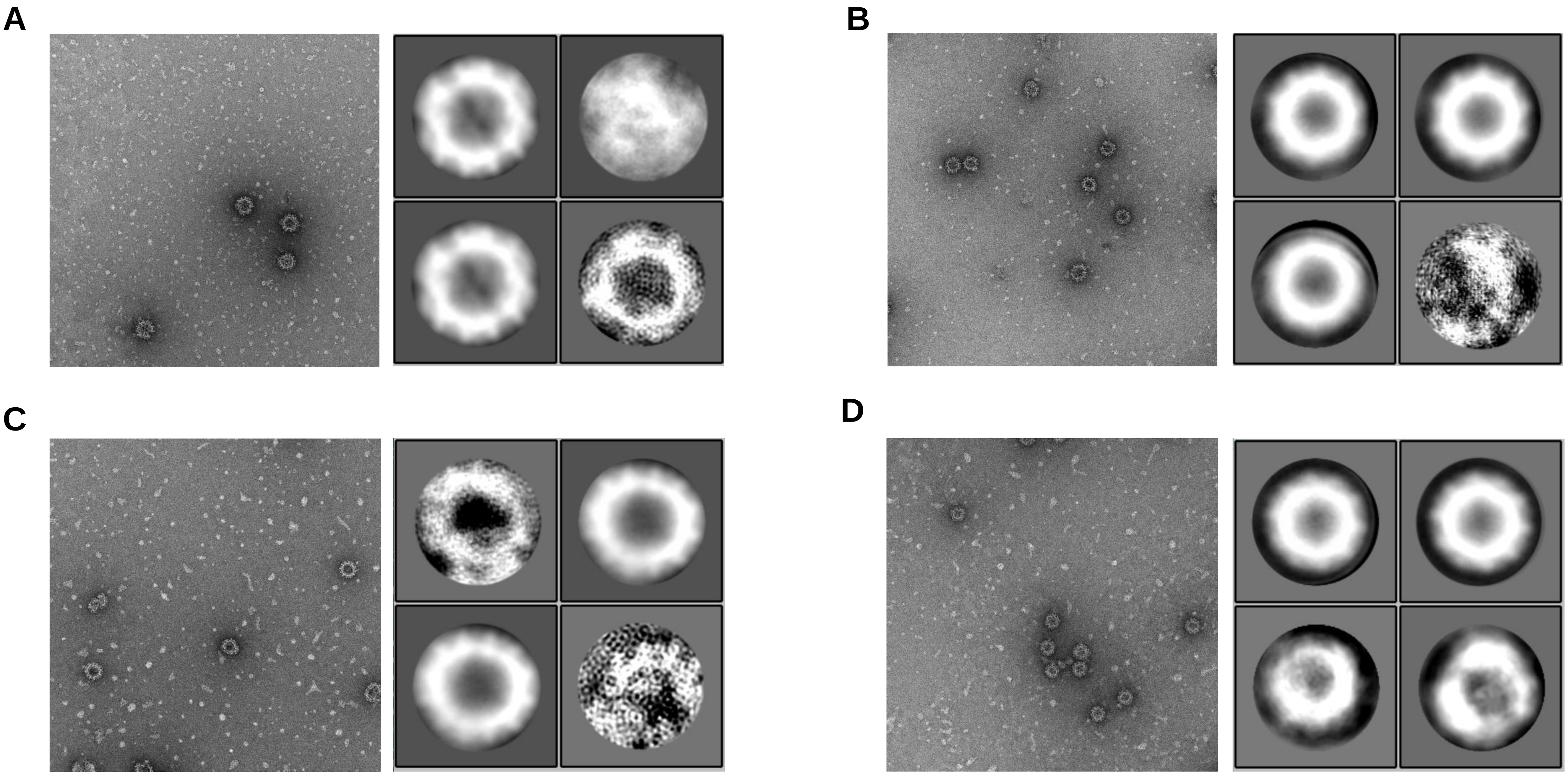

Supplemental Figure 3 - DS1 mutation leads to more uniform VLPs.
Representative negative-stain electron micrographs (nsEM) and 2D classes of GII.3 (**A**), GII.3-DS1 (**B**), GII.6 (**C**), and GII.6-DS1 (**D**) VLPs. 2D class averages of intact VLPs are in the panel set on the right. nsEM magnification is 58,000X. VLPs were stored at 4ºC until processed for imaging. **A** GII.3 2D class averages derived from 66 particles picked from 22 micrographs. **B** GII.3-DS1 2D class averages derived from 295 particles picked from 23 micrographs. **C** GII.6 2D class averages derived from 141 particles picked from 27 micrographs. **D** GII.6-DS1 2D class averages derived from 197 particles picked from 26 micrographs.

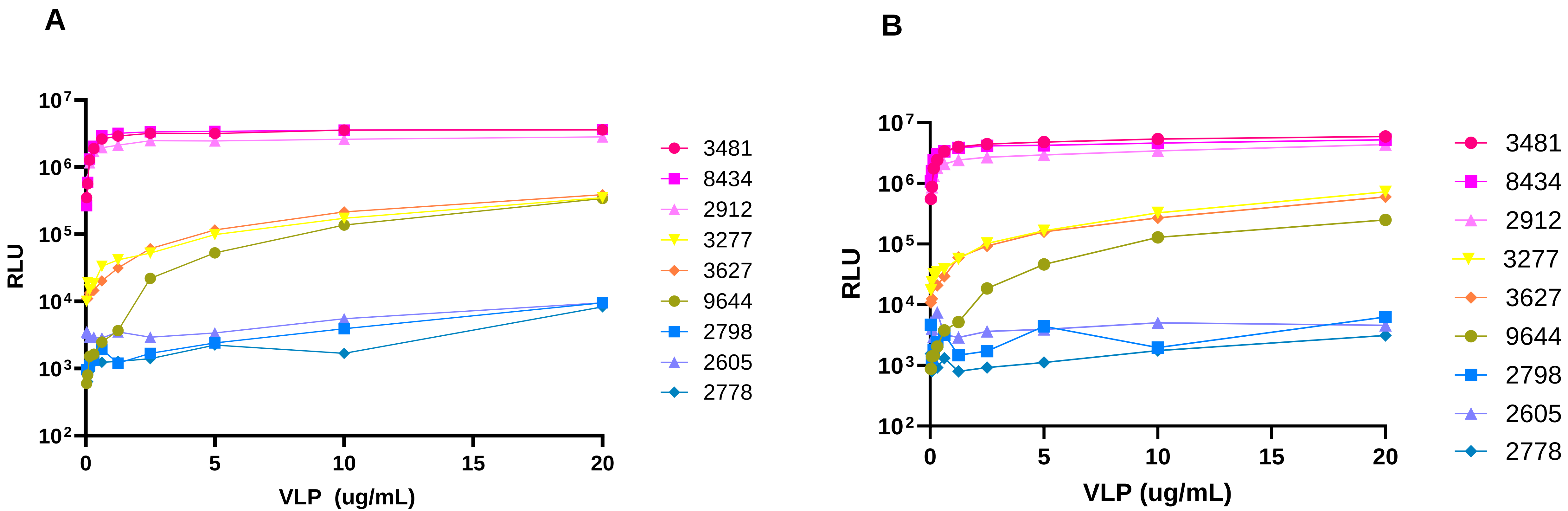

Supplemental Figure 4, GII.3 and GII.3-DS1 VLPs bind to human saliva samples. ELISA plates were coated with human saliva samples and incubated with either GII.3 (**A**) or GII.3-DS1 (**B**) VLPs in a two-fold serial dilution for 10 points starting from 20 µg/ml. Bound VLPs were detected using polyclonal sera against GII.3. The legend includes donor numbers for each individual saliva sample. Relative luminescent units (RLU) are plotted.

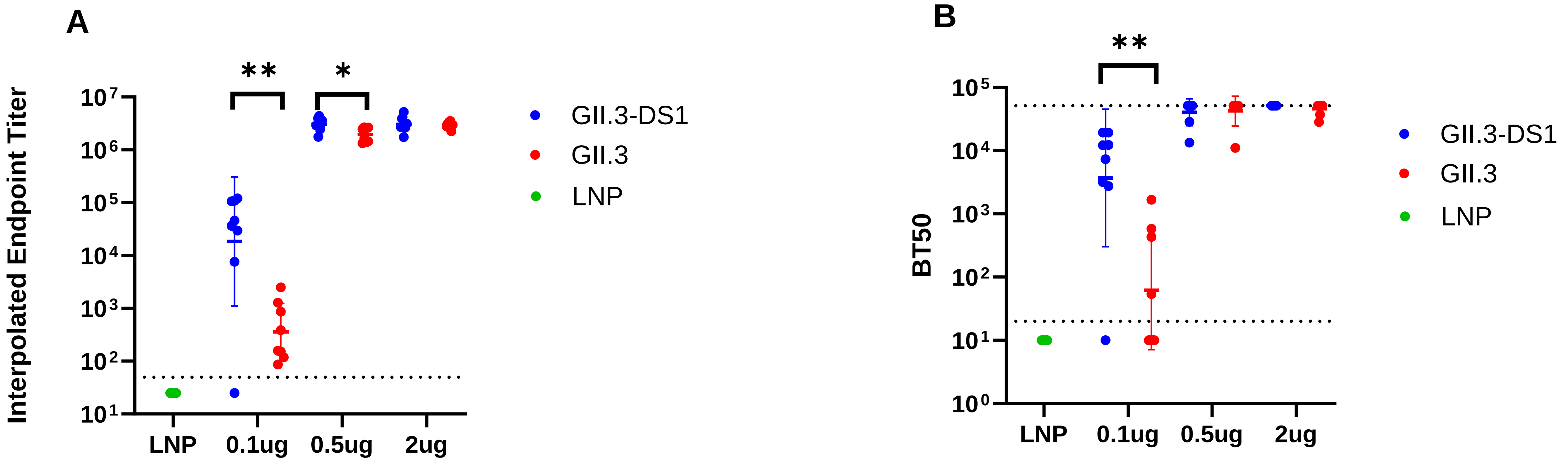

Supplemental Figure 5 - Mouse immunogenicity study of mRNAs encoding either GII.3 or GII.3-DS1, using assay reagent GII.3 VLP.
Week 6 sera shown in Fig VI were assayed for the presence of GII.3 binding antibodies (**A**) or HBGA blockade antibodies (**B**). Limits of detection are indicated with horizontal dotted lines (titer = 50 in **A** and 20 in **B**) . Empty lipid nanoparticle (LNP) was immunized as a negative control. Geometric mean titers with geometric standard deviation are shown in scatter dot plot. P values were determined using GraphPad Prism software ($p < 0.01$ = **, $p < 0.05$ = *).

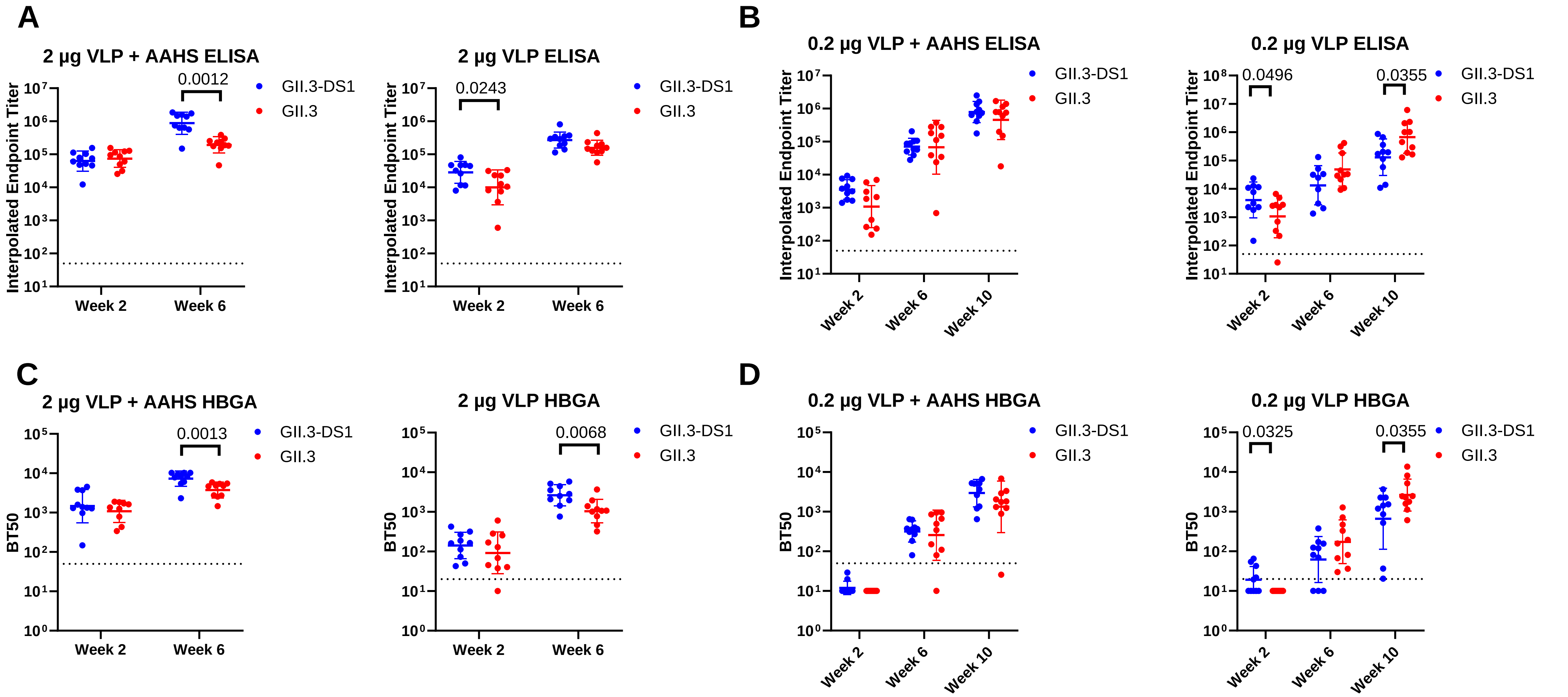

Supplemental Figure 6 - Mouse immunogenicity study of either GII.3 or GII.3-DS1 VLPs. BALB/c mice (N = 10 per group) were immunized with 2 ug (**A** and **C**) or 0.2 ug (**B** and **D**) of GII.3-DS1 (blue dots) or GII.3 VLPs (red dots) with and without AAHS at weeks 0 and 4. Blood was drawn at weeks 2, 6 and 10. Serum antibody ELISA titers (**A** and **B**) and HBGA blockade antibody titers (**C** and **D**) against GII.3-DS1 VLP were tested. Limits of detection are indicated with horizontal dotted lines (lower limit titer = 50 in **A** and **B** and 20 in **C** and **D**) . Geometric mean titers with geometric standard deviation are shown in scatter dot plots. P values were determined using GraphPad Prism software and P values less than 0.05 are shown.

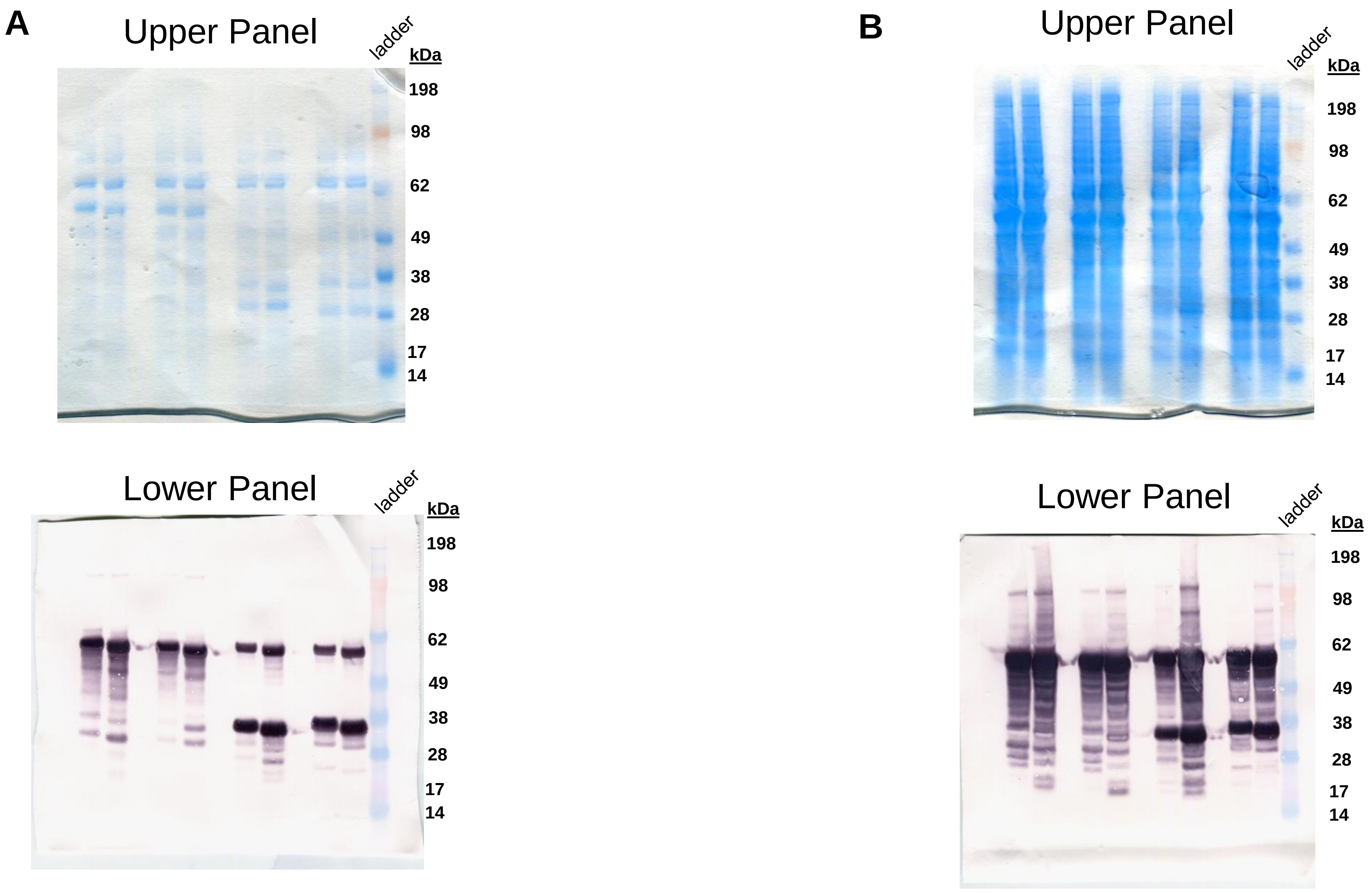

Supplemental Figure 7: Uncropped SDS-PAGE and western blot images related to main figure 2

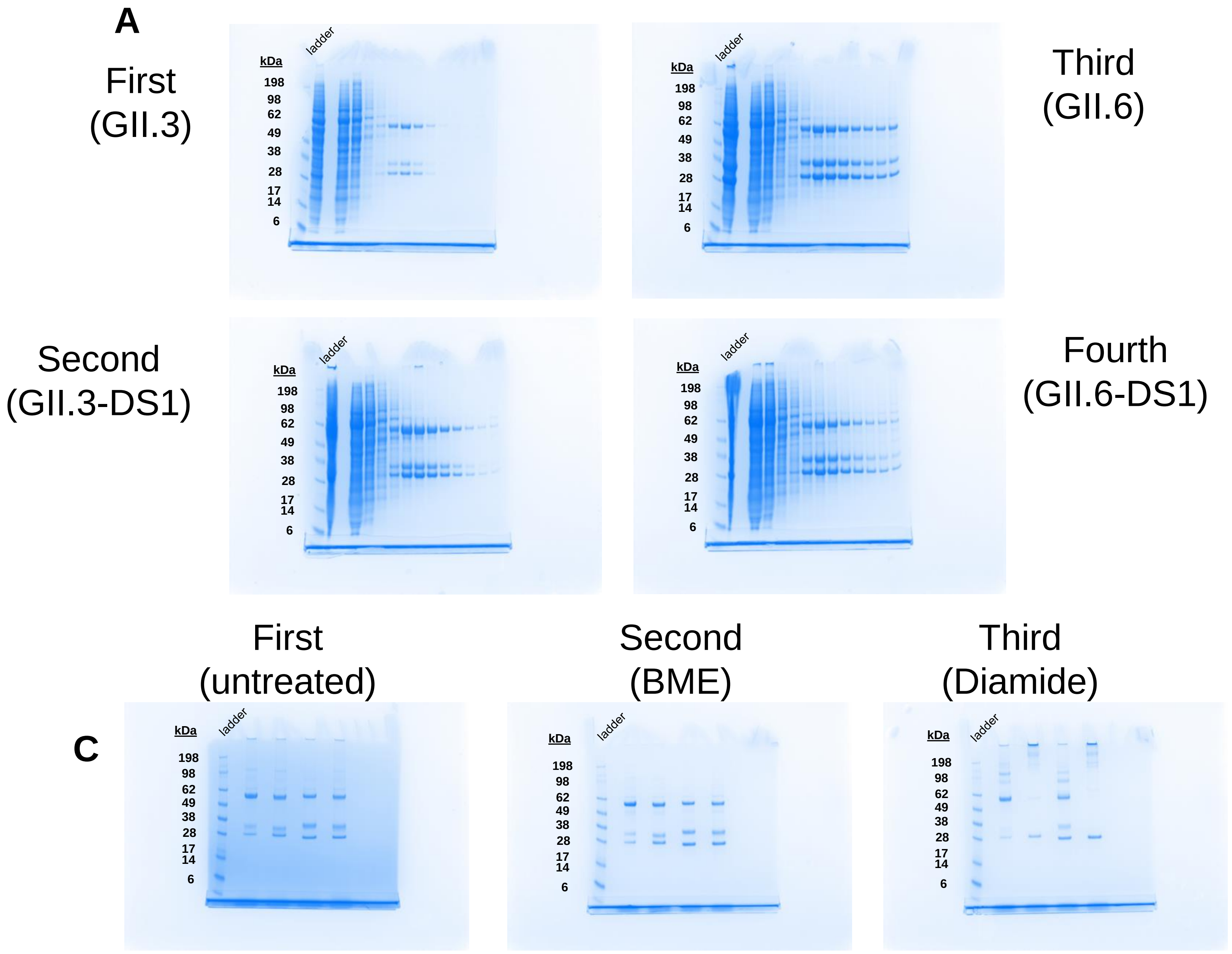

Supplemental Figure 8: Uncropped SDS-PAGE images related to main figure 3

A

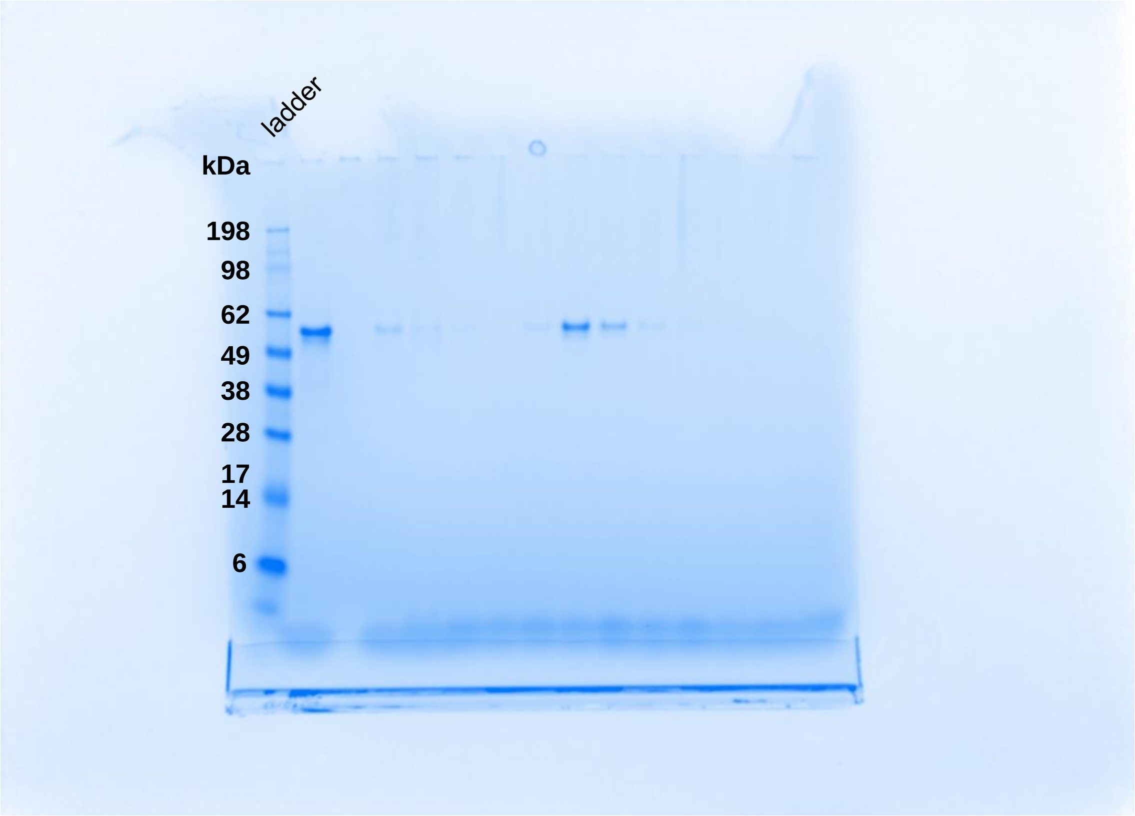

Supplemental Figure 9: Uncropped SDS-PAGE images related to supplemental figure 1

D

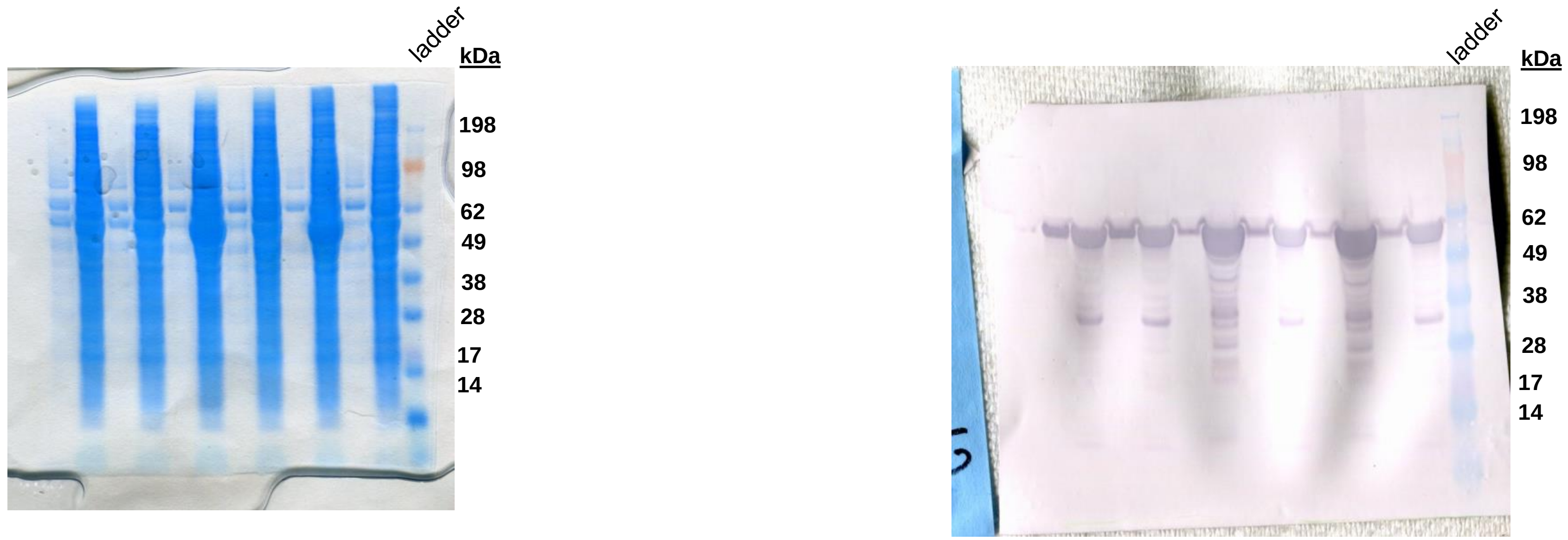

E

First (GII.3 D302A)

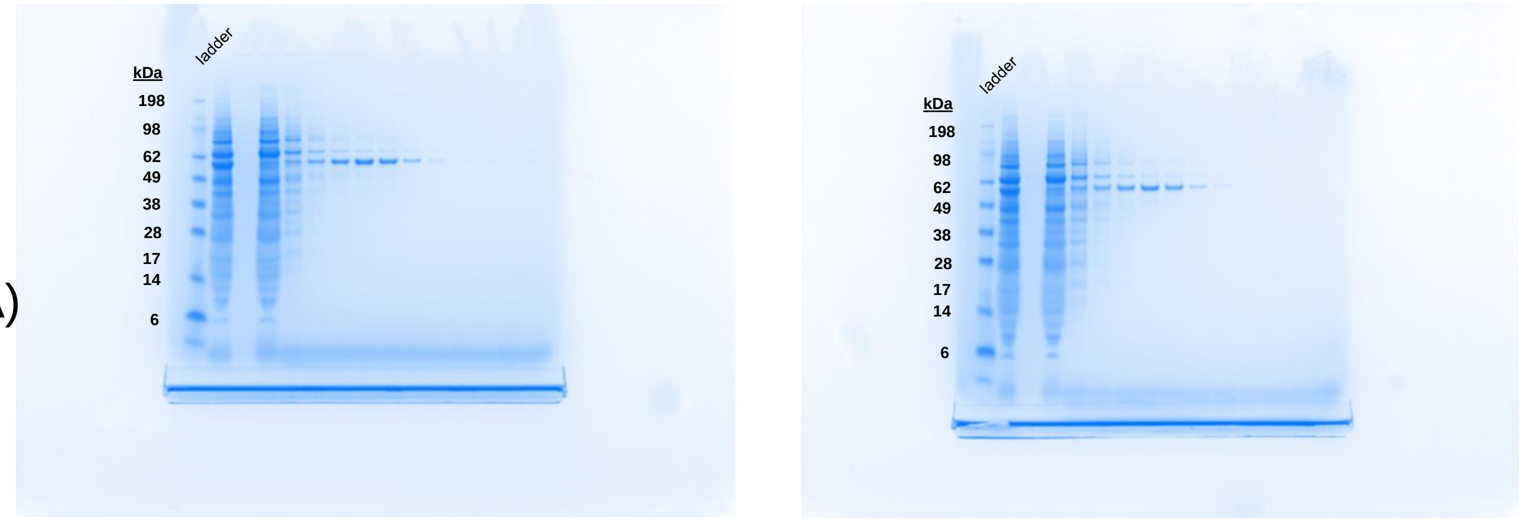

Second (GII.3 D302S)

Supplemental Figure 10: Uncropped SDS-PAGE and western blot images related to supplemental figure 2

Supplemental Table 1 – P Value Table

P values for the mouse immunogenicity studies in Figure 6 and Supplemental Figure 5.

**Figure 6**				
Panel	Assay	Week	Data Set	P Value
a	ELISA	2	0.1 ug	0.0079
a	ELISA	2	0.5 ug	0.0002
a	ELISA	2	2 ug	0.4089
a	ELISA	6	0.1 ug	0.0006
a	ELISA	6	0.5 ug	0.0541
a	ELISA	6	2 ug	0.2316
b	HBGA	2	0.1 ug	N/A
b	HBGA	2	0.5 ug	0.0002
b	HBGA	2	2 ug	0.0289
b	HBGA	6	0.1 ug	0.0020
b	HBGA	6	0.5 ug	0.4845
b	HBGA	6	2 ug	0.6126

**Supplemental Figure 5**				
Panel	Assay	Week	Data Set	P Value
a	ELISA	6	0.1 ug	0.0104
a	ELISA	6	0.5 ug	0.0140
a	ELISA	6	2 ug	0.6078
b	HBGA	6	0.1 ug	0.0044
b	HBGA	6	0.5 ug	N/A
b	HBGA	6	2 ug	N/A
